# CoMEt: a statistical approach to identify combinations of mutually exclusive alterations in cancer

**DOI:** 10.1186/s13059-015-0700-7

**Published:** 2015-08-08

**Authors:** Mark DM Leiserson, Hsin-Ta Wu, Fabio Vandin, Benjamin J. Raphael

**Affiliations:** Department of Computer Science, Brown University, 115 Waterman Street, Providence, 02912 RI USA; Center for Computational Molecular Biology, Brown University, Providence, Box 1910, 02912 RI USA; Department of Mathematics and Computer Science, University of Southern Denmark, Campusvej 55, Odense M Denmark

## Abstract

Cancer is a heterogeneous disease with different combinations of genetic alterations driving its development in different individuals. We introduce CoMEt, an algorithm to identify combinations of alterations that exhibit a pattern of mutual exclusivity across individuals, often observed for alterations in the same pathway. CoMEt includes an exact statistical test for mutual exclusivity and techniques to perform simultaneous analysis of multiple sets of mutually exclusive and subtype-specific alterations. We demonstrate that CoMEt outperforms existing approaches on simulated and real data. We apply CoMEt to five different cancer types, identifying both known cancer genes and pathways, and novel putative cancer genes.

**Electronic supplementary material:**

The online version of this article (doi:10.1186/s13059-015-0700-7) contains supplementary material, which is available to authorized users.

## Background

A major goal of large-scale cancer genomics projects such as The Cancer Genome Atlas (TCGA) [1–6], the International Cancer Genome Consortium (ICGC) [7, 8], and others is to identify the genetic and epigenetic alterations that drive cancer development. These projects have generated whole-genome/exome sequencing data measuring the somatic mutations in thousands of tumors in dozens of cancer types. Interpreting this data requires one to distinguish the *driver* mutations that play a role in cancer development and progression from *passenger* mutations that have no consequence for cancer. Identifying driver mutations directly from sequencing data is a significant challenge since individuals with the same cancer type typically harbor different combinations of driver mutations [9, 10].

The observed mutational heterogeneity in cancer has motivated the development of methods to examine *combinations* of mutations. Since driver mutations typically target genes in a small number of key pathways [11], several methods have been introduced to examine mutations in known pathways or networks (reviewed in [12, 13]). However, most pathway databases and interaction networks are incomplete, lack tissue specificity, and do not accurately represent the biology of a particular cancer cell. Thus, *de novo* methods for examining combinations of mutations are of particular interest as they require no prior biological knowledge and enable the discovery of novel combinations. Unfortunately, the number of possible combinations is too large to test exhaustively and achieve statistically significant results. Current *de novo* approaches to identify putative combinations of mutations use the observation that mutations in the same pathway are often mutually exclusive [14]. This observation follows from the observation that there are relatively few driver mutations in a tumor sample, and these are distributed over multiple pathways/hallmarks of cancer [15].

In 2011, three algorithms for identifying sets of genes with mutually exclusive mutations were introduced simultaneously: the De Novo Driver Exclusivity (Dendrix) [16], Recurrent Mutually Exclusive aberrations (RME) [17], and Mutual Exclusivity Modules (MEMo) [18] algorithms. Dendrix and RME are both *de novo* algorithms for identifying gene sets with mutually exclusive mutations, while MEMo examines mutual exclusivity on a protein-protein interaction network. The Dendrix algorithm identifies sets *M* of *k* genes with high coverage (many samples have a mutation in the set) and approximate exclusivity (few samples have a mutation in more than one gene in the set). Dendrix combines these two criteria into a weight *W*(*M*), which is equal to the coverage of *M* minus the coverage overlap (co-occurring mutations) of *M*. Finding the set of maximum weight is an NP-hard problem [16]. Dendrix uses a Markov chain Monte Carlo (MCMC) algorithm to sample high weight gene sets; more recently other optimization methods have been used to find high weight sets [19, 20]. Leiserson *et al*. [21] introduced the Multi-Dendrix algorithm to identify multiple mutually exclusive gene sets simultaneously using an integer linear program. In contrast, RME defines the exclusivity weight as the percentage of covered samples that contain exactly one mutation within a gene set, and uses an online-learning linear threshold algorithm to identify groups of genes with high pairwise exclusivity. However, both the RME and MEMo algorithms were shown not to scale to reasonably sized datasets [21], requiring extensive filtering of input data [17, 22].

**Fig. 1:**
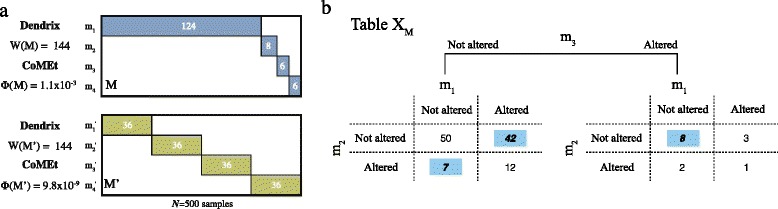
**a** Alteration matrices illustrating differences between the combinatorial weight function *W*(*M*) introduced in Dendrix and the probabilistic score *Φ*(*M*) used in CoMEt. Both matrices contain 4 mutually exclusive alterations whose alteration frequencies are indicated inside each bar. The samples without alterations are not shown in either matrix. Since both sets are exclusive and have the same total alteration frequency, the Dendrix weight function does not distinguish between these sets. Sets like *M* (blue) are common in cancer genome studies which often have a small number of recurrently mutated genes and a long tail of rarely mutated genes. The score used in CoMEt conditions on the observed frequencies of each alteration, giving more significance to the set *M* ^′^ (green). **b** An example of 2×2×2 contingency table **X**_*M*_ for the set *M*={*m*_1_,*m*_2_,*m*_3_}, illustrating how samples are cross-classified into exclusive, co-occurring, or absent for each alteration. The test statistic *ϕ*(*M*) used by CoMEt is the sum of the highlighted exclusive cells

One limitation of the combinatorial weight function used in Dendrix and subsequent algorithms is that genes with high mutation frequencies (high coverage) can dominate the mutual exclusivity signal, thus biasing the algorithms towards identifying gene sets where the majority of the coverage comes from one gene (Fig. 1(a)). These observations motivated the development of probabilistic models of mutual exclusivity. These include the Dendrix++ algorithm (an early version of the approach that we present in this paper) and the muex algorithm [23]. Dendrix++ uses a statistical score and was used in TCGA acute myeloid leukemia study [3]. The muex algorithm [23] uses a generative model of mutual exclusivity and a likelihood ratio test to score the mutual exclusivity of combinations of mutations. However, we find that the muex score is sensitive to high frequency mutations (see section Comparisons to other methods on real data). Moreover, both of these approaches exhaustively enumerate gene sets to find those with high score, limiting their applicability to larger datasets. In addition, they do not identify multiple gene sets simultaneously, a feature that has proved useful with the Dendrix weight [21]. The mutex algorithm [24] also uses a probabilistic model of mutual exclusivity, and was published after this manuscript was submitted. We provide further details of mutex below. Finally, no current method identifies overlapping gene sets^1^ — although cancer genes have been shown to participate in multiple pathways [1] — or considers additional sources of mutual exclusivity such as cancer subtype-specific mutations.

We introduce the Combinations of Mutually Exclusive Alterations (CoMEt) algorithm to overcome the challenges outlined above. CoMEt includes the following contributions.

1. We develop an exact statistical test for mutual exclusivity *conditional* on the observed frequency of each alteration. This approach is less biased towards high frequency alterations, and enables the discovery of combinations of lower frequency alterations. We derive a novel tail enumeration procedure to compute the exact test, as well as a binomial approximation.
2. CoMEt simultaneously identifies collections consisting of *multiple* combinations of mutually exclusive alterations, and samples from such collections using an MCMC algorithm. We summarize the resulting distribution by computing the marginal probability of pairs of alterations in the same sets. This enables CoMEt to identify sets of any size, including overlapping sets of alterations, without testing many parameter settings.
3. Given prior knowledge of cancer-types/subtypes, CoMEt analyzes alterations and subtypes simultaneously, allowing the discovery of mutually exclusive alterations across cancer types, while avoiding the identification of spurious mutually exclusive sets of (sub)type-specfic mutations.

We demonstrate that CoMEt outperforms earlier approaches on simulated and real cancer data. We apply CoMEt to acute myeloid leukemia (AML), glioblastoma (GBM), gastric (STAD), and breast cancer (BRCA) data from TCGA, and to a smaller study of intracranial germ tumors. In each cancer type, we identify combinations of mutated genes that overlap known cancer pathways and also contain potentially novel cancer genes including *IL7R* and the EphB receptor *EPHB3* in STAD, and the scavenger receptor *SRCRB4D* in GBM. On the gastric and breast cancer data, we demonstrate how CoMEt simultaneously identifies mutual exclusivity resulting from pathways and from subtype-specific mutations. CoMEt is available at [25] and as the cometExactTest R package available in CRAN [26].

## Results and discussion

### CoMEt algorithm

**Fig. 2:**
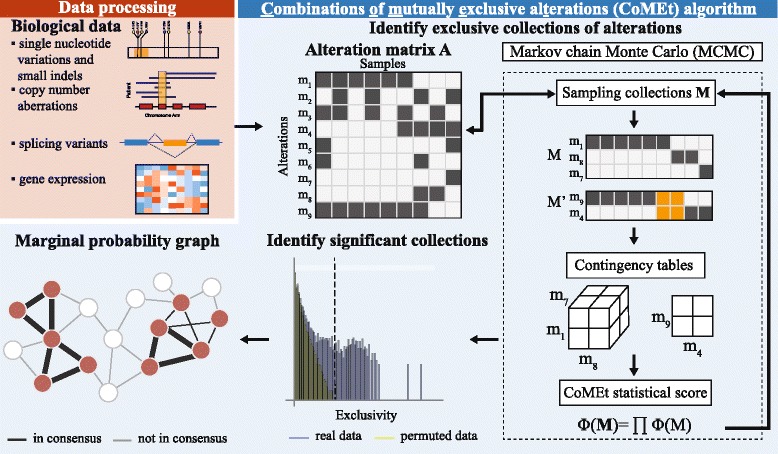
Overview of the CoMEt algorithm. First, we transform alteration data from different measurements into a binary alteration matrix *A*. Second, we use a Markov chain Monte Carlo (MCMC) algorithm to sample collections **M**, containing *t* sets of *k* alterations, in proportion to the weight *Φ*(**M**)^−*α*^. Here we show a collection containing sets *M* and *M* ^′^ with three and two alterations, respectively. We identify all collections whose weight exceeds the maximum observed in randomly permuted datasets. We summarize the alterations in these significant collections with a *marginal probability graph*, whose edge weights indicate the fraction of significant collections with the corresponding pair of alterations. Finally, we remove low-weight edges in the graph, obtaining the output modules

We consider that a set $\mathcal {E}$ of *malterations* have been measured in *n* samples. An alteration may be the somatic mutation of a particular gene, a specific single nucleotide mutation (for example, V600E mutations in the *BRAF* gene), an epigenetic change such as hypermethylation of a promoter, or a variety of other changes. We assume that alterations are binary, such that alterations are either present or absent in each sample. We represent the set of measured alterations with an *m*×*n* binary alteration matrix *A*=[*a*_*ij*_], where *a*_*ij*_=1 if alteration *i* occurs in sample *j*, and *a*_*ij*_=0 otherwise. Our goal is to identify *one or more* sets *M*_1_,*M*_2_,…,*M*_*t*_ where the alterations in each *M*_*i*_ are surprisingly mutually exclusive across the *n* samples. We introduce the CoMEt algorithm for this purpose (see Fig. 2), a preliminary version of which was presented at the RECOMB conference [27].

We derive a score *Φ*(*M*) for a set *M* of *k* alterations using an exact test of mutual exclusivity. Specifically, we examine a 2×2×⋯×2=2^*k*^ contingency table **X**_*M*_ (Fig. 1(b)) whose entries indicate the number of samples where each combination of alterations occurs. For example, the entry *x*_(24)_ of **X**_*M*_ equals the number of samples where the second and fourth alterations in *M* occur, but the first and third alterations do not occur. The score *Φ*(*M*) is the *P*-value of the observed mutual exclusivity in the table **X**_*M*_, where the margins of the table (determined by the number of samples where each alteration occurs) is fixed. That is, the score *Φ*(*M*) is *conditional* on the observed frequencies of alterations in *M*. This statistical score reduces the effect of the most frequent alterations having an unduly large contribution to the score. See section Materials and methods for further details.

CoMEt scores a collection **M**=(*M*_1_,…,*M*_*t*_) of *t* alteration sets by taking the product of the scores of each set *M*_*i*_:

(1) $$ \Phi(\mathbf{M}) = \prod_{i=1}^{t} \Phi(M_{i}).  $$

This score follows from the null hypothesis that exclusivity is independent across sets.

Since the number of possible collections of alteration sets grows exponentially with the number of alterations, it is typically impossible to enumerate and compute the weight of all alteration sets. We derive a Markov chain Monte Carlo (MCMC) algorithm to sample collections **M**, each consisting of *t* sets of alterations, in proportion to their significance. We summarize this distribution by computing the marginal probability *p*(*e*,*e*^′^) for each pair of alterations in *A*. We summarize these probabilities using the *marginal probability graph*, a complete, undirected weighted graph *G*=(*V*,*E*) where $V=\mathcal {E}$ (the set of observed alterations) and where each edge *e*∈*E* connects a pair of vertices *u*,*v* with weight *p*(*u*,*v*). We identify the most exclusive alteration sets by first removing all edges from the graph weight below a threshold *δ*. CoMEt outputs *C*(*δ*), the connected components in the resulting graph, which we call *modules*. The summarization via the marginal probability graph allows CoMEt to output collections of alteration sets different in number and size than specified by the input parameters. Further details are given in the section Materials and methods.

### Visualization of results

We created a web application for interactive visualization of the CoMEt results ([28]; see Additional file 1: Figure S1). For each dataset, the website shows the modules in the CoMEt marginal probability graph. Users can change the minimum edge weight parameter *δ*, which dynamically updates the modules. Edges in each module are labeled with the marginal probability. Users can view the rows of the alteration matrix that correspond to a given module, and also view, sort, and search through the collections sampled by CoMEt that include alterations in a given module.

### Comparison to other methods on simulated data

We compared CoMEt on two simulated mutation datasets to four other published methods for finding mutually exclusive gene sets: Dendrix [16], Multi-Dendrix [21], muex [23], and mutex [24]. In addition, we performed a separate comparison to MEMo [18] (see details in section Comparison to MEMo).

#### Benchmarking of methods for individual gene sets

We first compared the mutual exclusivity scores used by each of the methods on single gene sets using simulated datasets that represent key features of cancer sequencing data. In particular, each simulated dataset contains: (1) one implanted pathway *P* with *k*=3 genes that is altered in a fraction *γ*_*P*_ samples with highly exclusive mutations; (2) a set *C* of 5 highly altered genes whose alterations are not necessarily exclusive; (3) other genes containing only passenger mutations that were altered at rate *q*. The set *C* models the highly recurrently altered genes that often appear in real cancer datasets, and can confound methods for identifying exclusive mutations. Further details of the simulation are given in Additional file 1: Section Data.

We compared CoMEt to the other methods on datasets with *n*=500 samples and with implanted pathways with coverages *γ* ranging from 0.1 to 1.0. We ran CoMEt with *k*=3 for ten million iterations with 100 permutations, identifying modules in sets with *P*<0.05. We ran mutex with default parameters except with maximum group size set to 3 and with 1,000 permutations, and reported the gene sets above mutex’s suggested cutoff. We ran Dendrix and muex with *k*=3 and reported the highest scoring significant (*P*<0.05) set as neither algorithm outputs a consensus. We used coverage (parameter *γ* in [23]) and the weight *W* as the score for muex and Dendrix, respectively.

**Fig. 3:**
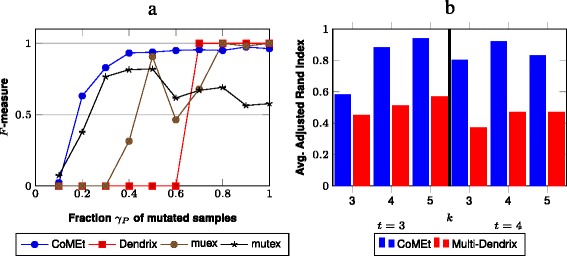
Comparison of CoMEt with other methods on simulated data with *n*=500 samples. **a** The average *F*-measure of each method over 25 simulated datasets with varying coverage of the implanted pathway: CoMEt (blue), mutex (black), muex (brown), and Dendrix (red). **b** Comparison of CoMEt and Multi-Dendrix in identifying an implanted *collection* containing multiple sets of alterations. Bars indicate average of adjusted Rand index between reported and implanted collection across 25 simulated datasets

We computed the precision and recall for each algorithm across 25 simulated datasets for each coverage *γ* (Additional file 2: Table S1). Wesummarized the results across the datasets using the *F*-measure, which is the harmonic mean of precision and recall. All the methods performed poorly (*F*<0.1) with coverage *γ*=0.1, and all the methods except mutex performed very well (*F*>0.9) for coverage *γ*≥0.8 (Fig. 3(a)). However, CoMEt outperformed the other methods for *γ*=0.2 to 0.6. Both muex and Dendrix struggled to identify the implanted pathway (*F*<0.4) with coverage *γ*<0.5. In comparison, CoMEt had *F*≥0.6 for *γ*>0.2. While mutex’s performance was only slightly below that of CoMEt with *γ*<0.5, mutex performed poorly compared to CoMEt and the other methods with *γ*≥0.6. Interestingly, the reason mutex performed poorly is because it identified many false positives resulting in a low precision (≤0.6) even though its recall was 1.0. These false positive gene sets often include at least one gene from *C* (the set of highly altered genes), indicating a problem with mutex’s mutual exclusivity score. These simulations demonstrate the advantages of CoMEt’s mutual exclusivity score in identifying mutually exclusive sets of genes (even when rarely mutated) in the presence of highly altered genes.

#### Benchmarking identification of collections of gene sets

We compared CoMEt to Multi-Dendrix [21], an earlier method that also simultaneously finds collections containing more than one mutually exclusive set. We compared these two algorithms on two types of simulated datasets: one containing collections of gene sets with no overlapping genes, and the other containing overlapping gene sets. We generated simulated data using a procedure similar to that above with three important differences. First, we implanted a collection **P**=(*P*_1_,*P*_2_,…,*P*_*t*_) of *t* pathways, each with exclusive mutations with total coverage *γ*_**P**_. Second, all genes in each implanted pathway are mutated in the same number of samples. Third, we include *m*=20,000 genes and remove those mutated in fewer than 1 *%* of total samples (Additional file 1: Figure S2). We generated datasets varying *t* from 2 to 4 and *k* from 3 to 5 with coverages *γ*_*P*_ between 0.40 and 0.70 (Additional file 2: Table S2). We also generated datasets with *overlapping* implanted pathways with *t*=3, *k* from 3 to 5, with *γ*_**P**_=(0.75,0.75,0.60).

On each dataset, we ran CoMEt using *k*=4,*t*=3, and Multi-Dendrix using its default parameters of *t* ranging from 2 to 4, and *k* ranging from 3 to 5. We compared the consensus sets output by Multi-Dendrix with the modules output by CoMEt, using the adjusted Rand index (ARI) [29], to score how well each algorithm identified the implanted pathways. The ARI measures the agreement between two partitions, with ARI = 1 indicating that two partitions are identical and ARI = –1 indicating that two partitions are maximally dissimilar. CoMEt outperformed Multi-Dendrix in 11/12 simulated datasets (each containing 25 replicates) (Fig. 3b and Additional file 2: Table S3). CoMEt found a much larger fraction of the implanted pathways (difference in ARI was >0.2 for 8/12 datasets). Furthermore, CoMEt had an ARI >0.5 for all 12 datasets, and ARI >0.8 for 7/12 datasets. We emphasize that we ran CoMEt with a *single* value of *t* and a *single* value of *k* over all datasets even though the size and number of implanted pathways varied across datasets. In contrast, Multi-Dendrix was run with a range of parameter values. This demonstrates that CoMEt is much less sensitive to parameter choices than Multi-Dendrix.

We also compared the output of CoMEt and Multi-Dendrix using the true values of *t* and *k*. We found that CoMEt outperformed Multi-Dendrix on 11/12 datasets (Additional file 2: Table S3). This shows that the statistical score used by CoMEt and the MCMC sampling are important features, even on simulated datasets where the implanted collections are fairly strong signals in the data.

### CoMEt results on real cancer datasets

We ran CoMEt on four mutation datasets from TCGA: glioblastoma (GBM) [1], breast cancer (BRCA) [4], gastric cancer (STAD) [6], and acute myeloid leukemia (AML) [3]. We also analyzed the dataset of intracranial germ tumors from Wang *et al.* [30]. Because CoMEt can analyze any type of binary alterations, we include many types of alterations in these datasets: small indels and single nucleotide variations, copy number aberrations, aberrant splicing events, gene fusions, and (for BRCA and STAD) cancer subtype. See section Somatic mutation datasets for details on these datasets and Additional file 1: Section Methods for details on parameters.

**Acute myeloid leukemia (AML)** We first ran CoMEt with *t*=4 alteration sets, each of size *k*=4. The CoMEt output contains four mutually exclusive modules that include 18 alterations (Additional file 1: Figure S3). These four modules are: (1) *TP53*, *RUNX1*, *NPM1*, *PML-RAR α* (52.5 *%* of samples); (2) *KDM6A*, *FLT3*, tyrosine kinases, *RAS* proteins, serine/threonine kinases, *DNMT3A*, *MLL*-X fusions, *MYH11-CBF β*, and *RUNX1-RUNX1T1* fusion (70 *%* of samples); (3) cohesin complex, other myeloid transcription factors, and other epigenetic modifiers (33 *%* of samples); (4) *TET2* and *IDH2* (18.5 *%* of samples).

**Fig. 4:**
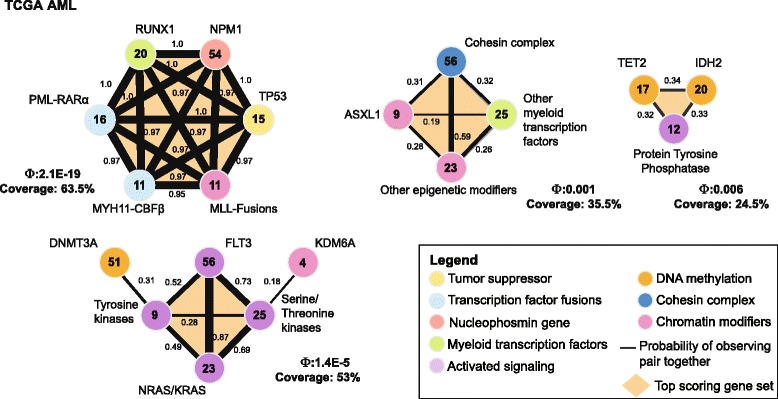
CoMEt results on TCGA AML consisting of four modules. Each circle represents the alterations in a gene or genomic region. The number in the circle indicates the number of samples in which the alteration occurs. Black lines are edges in the marginal probability graph with indicated probabilities. Orange polygons indicate the sets in the collection **M** with the most significant value *Φ*(**M**). Below each most significant set (orange) are the corresponding score *Φ* and coverage

The recent TCGA AML publication [3] reported strong mutual exclusivity (using an earlier version of the CoMEt algorithm, called Dendrix++) across several expert-defined classes. Thus, we increased the value of *k* to identify *t*=4 gene sets with sizes *k*=6,4,4,3. Because of the larger values of *k*, we increased the number of MCMC iterations to 200 million (Additional file 2: Table S4). The resulting marginal probability graph (*δ*=0.179) contained four mutually exclusive modules with a total of 19 genes (Fig. 4).

The first module contains six perfectly mutually exclusive alterations. These six alterations include: mutations in *TP53*, *RUNX1*, *NPM1*; *PML-RAR α*, *MYH11-CBF β* fusion genes, and other *MLL* fusions, which we denote as *MLL*-X fusions, following [3]. These six alterations are known to be drivers in AML, and together are found in 63.5 *%* of the samples. These fusion genes are defining aberrations for certain subtypes of AML, as *PML-RAR α*, *MYH11-CBF β*, and *MLL* fusions are associated with acute promyelocytic leukemia, acute monoblastic or monocytic leukemia, and acute megakaryoblastic leukemia, respectively. The second module (altered in 63 *%* of samples) contains receptor tyrosine kinases (RTKs) and their downstream RAS target proteins. These include mutations in the *FLT3* tyrosine kinase, other tyrosine kinases, serine/threonine kinases, and *RAS* proteins. Two additional genes, *DNMT3A* and *KDM6A*, are also included in this set. These genes are involved in DNA/histone methylation, and their interactions with the other RTK/RAS genes in the set are less clear. Notably, the marginal probability graph (Fig. 4) shows that the connection between *DNMT3A* and other genes in the set is largely due to its mutual exclusivity with other tyrosine kinases, and in fact a number of samples have mutations in both *FLT3* and *DMNT3A* (Additional file 1: Figure S4). Thus, the patterns of exclusivity/co-occurrence between alterations may be subtle, demonstrating the advantages of CoMEt’s approach to simultaneously examine multiple collections of sets of alterations.

The third module (altered in 35.5 *%* of samples) contains genes related to chromatin modification and gene regulation including *ASXL1*, the cohesin complex, other myeloid transcription factors, and other epigenetic modifiers. Finally, the fourth module (altered in 24.5 *%* of samples) contains genes related to DNA methylation including *TET2*, *IDH2*, and protein tyrosine phosphatases. Mutual exclusivity between *TET2* and *IDH2* in AML has been previously reported [31–33]. Moreover, recent work provides a mechanistic explanation for this observed exclusivity: Figeroa et al. [31] show that mutant *IDH12* inhibits *TET2*’s function in demethylation of 5-methylcytosine. These results demonstrate that CoMEt is able to extract multiple functional modules directly from alteration data.

**Fig. 5:**
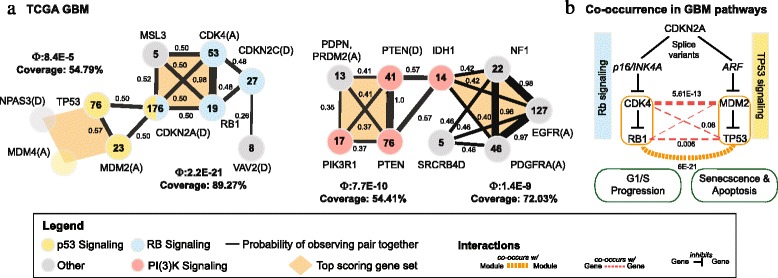
CoMEt results on TCGA GBM. **a** Two output modules from CoMEt are shown in the same style as in Fig. 4. Characters in parentheses following gene name indicate copy number aberrations: (D) is a deletion, and (A) is an amplification. **b** Different splice variants of *CDKN2A* are part of both the Rb signaling (left) and p53 signaling (right) pathways. CoMEt recovers this relationship as two separate mutually exclusive gene sets. The gene sets {*RB1*, *CDK4*} and {*MDM2*, *TP53*} have a statistically significant number of co-occurring mutations (*P*=6×10^−21^, dotted orange line), which is much more significant than the co-occurrence between pairs of genes in these sets (dotted red lines with corresponding *P*-values)

**Glioblastoma multiforme (GBM)** We ran CoMEt on the TCGA GBM dataset from Leiserson *et al*. [21] with *t*=4 and *k*=4 (Additional file 2: Table S5). While Leiserson *et al.* [21] removed amplifications in *EGFR* because they were so frequent it confounded their analysis, we added these amplifications back when running CoMEt, treating *EGFR* amplifications and *TP53* as subtypes so they could not be sampled in the same set (see section Simultaneous analysis of alterations and cancer subtypes for details). The resulting marginal probability graph (*δ*=0.263) includes two mutually exclusive modules (Fig. 5(a)).

The first module includes alterations in three genes in the Rb signaling pathway (*CDK4*, *RB1*, *CDKN2C*) and in three genes in the p53 signaling pathway (*TP53*, *MDM2*, and *MDM4*), as annotated by the original TCGA GBM publication [1]. This module also contains deletions in *CDKN2A*, which is a member of both the Rb signaling and p53 signaling pathways. Indeed, it is well known that different isoforms of the *CDKN2A* gene are involved in the Rb and p53 signaling pathways (see Fig. 5(b) and also [1]) and that the genomic deletion of *CDKN2A* affects both isoforms. Moreover, we find that the pairs *CDK4*-*RB1* and *MDM2*-*TP53* have surprisingly co-occurring alterations (*P*=6×10^−21^; see Fig. 5(b)). This co-occurrence is stronger than the co-occurrence of alterations in individual genes. This pattern indicates that glioblastomas can alter the function of the Rb and p53 signaling pathways either by deleting *CDKN2A*, *or* by altering one gene in each of the pairs (*CDK4*, *RB1*) and (*TP53*, *MDM2*). We emphasize that CoMEt identified this overlapping module by sampling *nonoverlapping* exclusive sets. Finally, this module contains alterations in three additional genes: *NPAS3*, *VAV2*, and *MSL3*. *NPAS3* has been studied as a novel late-stage acting progression factor in gliomas with tumor suppressive functions [34, 35]. *VAV2* has been reported to regulate *EGFR*, and knockdown of *VAV2* enhanced EGFR degradation and further reduced cell proliferation [36]. MSL3 is a member of the male-specific lethal (MSL) complex and is thought to play a role in transcriptional regulation. As reported in [21], the MSL complex also includes MOF, which regulates p53 in the cell cycle and may be involved in cancer [37].

The second module includes alterations in the PI(3)K signaling pathway — including *PIK3R1*, *PTEN*, deletion of *PTEN* and *IDH1* — as well as amplifications in the genes (*EGFR*, *PDGFRA*) and in a region containing *PRDM2* and *PDPN*. Additional genes in this module are *NF1* and *SRCRB4D*. The PI(3)K signaling pathway genes overlap the results reported by Multi-Dendrix on this dataset in [21]; the important differences are that CoMEt includes *NF1* and amplifications in *EGFR* (which were not analyzed by [21]). In this module, we also found one mutually exclusive gene set (from the highest weight collection) that includes *EGFR*, *IDH1*, *NF1*, and *PDGFRA*. Alterations in these genes have strong association with individual subtypes in GBM [38]: *EGFR* amplification is associated with the Classical GBM subtype, *IDH1* and *PDGFRA* amplification are associated with the Proneural GBM subtype, and *NF1* is associated with the Mesenchymal GBM subtype. This shows that mutually exclusive gene sets can result from subtype-specific mutations.

Finally, *SRCRB4D* is a scavenger receptor with no known associations with cancer. However, two other scavenger receptor genes have previously reported roles in glioblastoma. Homozygous deletions of *DMBT1* were reported in glioblastomas and astrocytomas [39, 40]. *CD36* was recently reported to be involved in cancer stem cell maintenance in glioblastoma [41].

These results show that CoMEt can automatically find large portions of the pathways that were manually curated in TCGA GBM publication [1], including overlapping pathways. Moreover, CoMEt identifies additional genes with putative roles in glioblastoma and significant exclusivity with other known glioblastoma genes.

**Gastric cancer (STAD)** We performed two runs of CoMEt on the TCGA gastric cancer (STAD) dataset, and then merged the runs (described in section Simultaneous analysis of alterations and cancer subtypes). We first ran CoMEt with *t*=4 and *k*=4. We then ran CoMEt on a STAD dataset that included sample subtype classifications. TCGA recently classified gastric cancers into four subtypes based on integration of different molecular data [6]. To examine the relationships between subtypes and other alterations, we introduce “subtype alterations” for the three subtypes from [6] (we did not include the hypermutated samples from the MSI subtype in our analysis). As described in section Simultaneous analysis of alterations and cancer subtypes, these “subtype alterations” are marked as altered in samples that are *not* members of the subtype, so that mutual exclusivity between a “subtype alteration” and another alteration indicates that the alteration is enriched in the subtype. We ran CoMEt on the STAD dataset with subtype alterations using *k*=4 and *t*=3 (the number of subtypes).

**Fig. 6:**
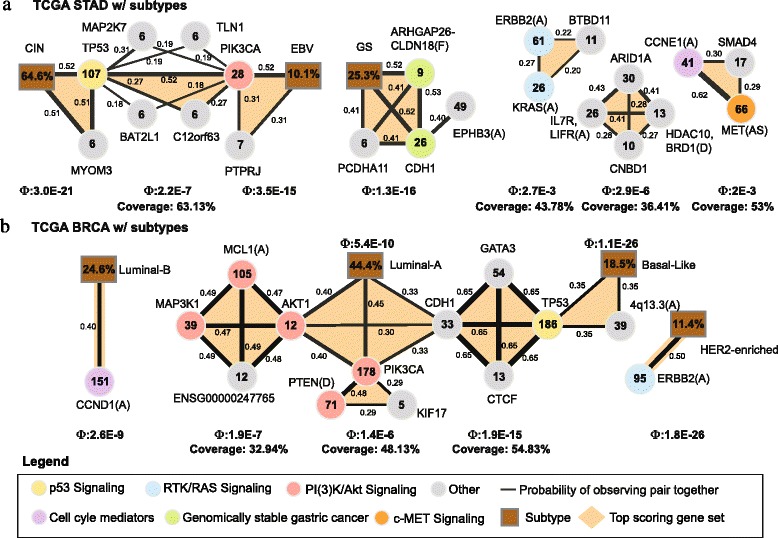
CoMEt results on (**a**) TCGA STAD subtypes, (**b**) TCGA BRCA subtypes. Style is the same as in Fig. 5, except for the addition of subtype alterations (brown) and additional characters in parentheses following gene name: **(AS)** is an alternative splicing event, and **(F)** is a fusion gene. Note that an edge between a subtype (brown vertex) and an alteration indicates that the alteration occurs frequently in the subtype

CoMEt identified five mutually exclusive modules from the marginal probability graph (*δ*=0.132) in the STAD dataset (Additional file 2: Table S6 and Fig. 6(a)). Each of these modules includes known cancer genes and novel candidate genes. Two modules indicate subtype-specific altered genes and pathways. The first module (altered in 69 % (150/217) of the STAD samples) includes two genes, *TP53* and *PIK3CA*, that are enriched for alterations in the CIN and EBV subtypes, respectively. TCGA gastric study reported that 80 *%* of EBV tumors contain an alteration in *PIK3CA*, and suggested that EBV tumors might respond to PI3-kinase inhibitors [6]. Given this strong signal, it is not surprising that these two genes appear in CoMEt results. However, these signals do not dominate the CoMEt results, and four other interesting modules are also output. There are six other mutated genes in this first module including *MAP2K7*, *TLN1*, *BAT2L1*, *C12orf63* (recently renamed *CFAP54*), *MYOM3*, and *PTPRJ*. Given the rarity of these mutations, their significance is unclear.

The second STAD model includes the genomically stable (GS) subtype, mutations in *CDH1*, mutations in *PCDHA11*, *ARHGAP6-CLDN18* fusions, and amplification of a region containing *EPHB3*. *CDH1* somatic mutations and *ARHGAP6-CLDN18* fusions were reported to be mutually exclusive and enriched in the genomically stable subtype in gastric cancer [6], and CoMEt recapitulates this result. *EPHB3* is the member of Eph/ephrin signaling which controls the compartmentalization of cells in epithelial tissues. A recent study [42] demonstrated EphB receptors (for example, *EPHB1* and *EPHB3*) interacting with *CDH1* in epithelial intestinal cells which regulates the formation of E-cadherin-based adhesions. This interaction explains the perfect mutual exclusivity between *CDH1* and *EPHB3*, which to our knowledge is the first report of this relationship. This demonstrates that mutual exclusivity between pairs of alterations/subtypes may have subtle explanations, further underscoring the need for analysis of collections of multiple alterations.

The third module (altered in 95/217 of samples) includes amplifications of *KRAS* and *ERBB2*, and mutations in *BTBD11*. *KRAS* and *ERBB2* are members of the RTK/RAS signaling pathway, and their role in cancer is well-documented. Little is known about the function of *BTBD11*, and thus the significance of the mutations is unclear.

The fourth STAD module (115/217 of samples) contains three altered genes, including amplifications of *CCNE1*, mutations in *SMAD4* and splice-site mutations in *MET*. *CCNE1* is a well-known cell cycle mediator, *SMAD4* is a member of the TGF- *β* pathway, and *MET* participates in the RTK/RAS signaling pathway [6].

The fifth STAD module (79/217 of samples) contains four altered genes, including amplifications in a region with *IL7R* and *LIFR*, deletions in a region with *HDAC10* and *BRD1*, mutations in *ARID1A*, and mutations in *CNBD1*. *ARID1A* is a well-known cancer gene shown to be significantly mutated in gastric cancer [6]. Moreover, inhibition of *HDAC10* has been reported to be associated with human gastric cancer cells [43]. Gain-of-function mutations in *IL7R* have been reported to be associated with childhood acute lymphoblastic leukemia [44]. Our CoMEt results suggest that *IL7R* mutations may have a role in gastric cancer as well.

**Breast cancer (BRCA)** We performed two runs of CoMEt on the TCGA breast cancer (BRCA) dataset, and then merged the runs. We first ran CoMEt with *k*=4 and *t*=3. We then introduced subtype alterations for four subtypes from [4] (as described in section Simultaneous analysis of alterations and cancer subtypes). Breast cancers are traditionally classified into multiple subtypes based on mRNA expression. Here we analyze four subtypes: luminal A, luminal B, basal-like, and HER2-enriched. We ran CoMEt on a BRCA dataset that included sample subtype classifications with *k*=4 and *t*=4 (the number of subtypes).

CoMEt identified three subtype-specific modules and three modules with mutated genes (Additional file 2: Table S7 and Fig. 6(b)) in the marginal probability graph (*δ*=0.287). The first module shows the strong association between amplification of *CCND1* and the luminal B subtype as previously reported [45]. Similarly, the third module shows the strong association between *ERBB2* amplification and the HER2 (*ERRB2*)-enriched subtype.

The second module shows a complicated relationship between: (1) subtype-associated alterations in the luminal A and basal-like subtypes, and (2) mutual exclusivity resulting from alterations in the same pathway(s). This module contains five sets of genes (highlighted in orange in Fig. 6(b)) in the highest scoring collection **M** output by CoMEt. Consistent with TCGA study [4], we find that *CDH1*, *AKT1*, and *PIK3CA* are associated with the luminal A subtype, and they form a set in the CoMEt output. Similarly, *TP53* and amplification of chromosome region 4q13.3 are associated with the basal-like subtype, and they also form a set in the CoMEt output. Two of the other sets contains genes in the same pathway. *PTEN* is a known inhibitor of *PIK3CA*, explaining the observed exclusivity between *PTEN* deletion and *PIK3CA* mutation. Moreover, *MCL1*, *MAP3K1*, *AKT1* are all part of the PI(3)K/Akt signaling pathway. Together, these sets contain five genes that are annotated as part of the PI(3)K/Akt signaling pathway in TCGA study [4] (red circles in Fig. 6(b)).

The final set in this module includes mutations in the genes *TP53*, *CDH1*, *GATA3*, and *CTCF*. These four genes are altered in 54.83 *%* (278/507) of the BRCA samples. *TP53* is a member of the p53 signaling pathway, while *CDH1*, *GATA3*, and *CTCF* all have been reported as potential driver genes in breast cancer. *CDH1* is a tumor supressor that is well-known to play multiple roles in cancer [46], including invasion and proliferation in breast cancer [47]. *GATA3* is a transcription factor that has long been known to be involved in breast cancer tumorigenesis [48]. Recently, *GATA3* has been reported to promote differentiation, suppress metastasis, and alter the tumor microenvironment in breast cancer [49]. As noted by Leiserson *et al.* [21], *GATA3* has also been reported to suppress tumor metastases through inhibition of *CDH1* promoters [50], which suggests that the mutations in *GATA3* are an alternate way to downregulate *CDH1* and may explain the exclusivity of the mutations in *GATA3* and *CDH1*. Moreover, *GATA3* is enriched for mutations in both luminal A and luminal B; that is, 32 of the 54 mutations in *GATA3* occur in luminal A (*P*=0.0207) and 19 of the 54 mutations in *GATA3* occur in luminal B (*P*=0.065). This might suggest that *GATA3* mutations mainly occur in patients with luminal breast cancer. *CTCF* neighbors *CDH1* on chromosome 16q22.1 and has been reported with *CDH1* to be a tumor suppressor in breast cancer [51, 52]. Interestingly, both *CDH1* and *CTCF* have most of their mutations in samples of the luminal A subtype. *CDH1* is enriched for mutations in luminal A (as reported in [4]) and 9 of the 13 mutations in *CTCF* occur in luminal A (*P*=0.0891), suggesting that these two genes are in a pathway specifically targeted in luminal A. Furthermore, 4 of the 9 mutations in *CTCF* in luminal A are missense mutations in zinc finger domains, suggesting a possible functional role for these mutations [53].

Together, these results demonstrate CoMEt’s ability to simultaneously identify alterations that are mutually exclusive due to interactions between genes in pathways or due to subtype-specific alterations. This allows a more refined interpretation of mutually exclusive alterations than simple pairwise analyses.

**Intracranial germ tumors** To investigate CoMEt’s performance on a smaller dataset that is less intensively studied than TCGA datasets, we ran CoMEt on a dataset of somatic and germline mutations in intracranial germ cell tumors (IGCTs) from [30]. This dataset consists of somatic single nucleotide variants and indels in 163 genes from 53 patients (combining both the discovery and validation cohorts). Given the small size of this dataset, we first ran CoMEt to identify *t*=1 set of *k*=3 genes (Additional file 2: Table S8). CoMEt found that the alterations in the set of *k*=3 genes *KIT* (16 mutations), *KRAS* (9), and *NRAS* (3) were the most exclusive (*Φ*=0.002). Wang *et al.* [30] identified this triple using Fisher’s exact test comparing mutations *KIT* with the union of mutations in *KRAS* and *NRAS* (*P*=0.018). Notably, the exact test gives the triple a more significant *P*-value. Mutual exclusivity between these three genes is consistent with the RAS genes being downstream of *KIT* in the signaling receptor tyrosine kinase (RTK) signaling pathway.

The top-ranked *KRAS*, *NRAS*, *KIT* triple was closely followed by several other gene sets including *KIT*, *KRAS*, and a third gene (*FLT3*, *Φ*=0.004; *KDM2A*, *Φ*=0.004; *LAMA4*, *Φ*=0.004; *SPRY4*, *Φ*=0.004). Notably, *KIT* and *FLT3* (2 mutations) are both receptor tyrosine kinases (RTKs); the mutual exclusivity of their mutations suggests that *FLT3* mutations may substitute for *KIT* mutations in some samples. In addition, *SPRY4* is a negative regulator of RAS signaling and was recently shown to inhibit RAS signaling in AML [54]. *SPRY4* was not discussed in the Wang *et al.* study, and thus is a novel discovery by CoMEt. Intriguingly, the observed mutual exclusivity that we see in the high-scoring gene triples from CoMEt (Additional file 2: Table S8) are similar to relationships seen between RTK and RAS signaling in AML [3, 54].

CoMEt summarized the mutually exclusive sets into two statistically significant modules (*P*<0.01). The first module includes *KIT*, *KRAS*, *NRAS*, *TP53*, and *LAMA4* (Additional file 1: Figure S5), which are collectively mutated in 62 % (33) of the 53 patients. All five of these genes were identified as containing significantly recurrent mutations by Wang *et al.* The second module contains perfectly exclusive mutations in *JMJD1C* and *CBL*, which are mutated in 30 % (16) of the 53 patients. *CBL* is the third most somatically mutated gene in the Wang *et al.* study, and Wang *et al.* described a role for *CBL* as a negative regulator of RTKs, including *KIT*. However, mutations in *CBL* and *KIT* are not significantly exclusive (*P*=*Φ*=0.253). This is because *CBL* is mutated in only six samples, one of which also has a mutation in *KIT*. Furthermore, the exclusivity between mutations in the gene triple, *KIT*, *KRAS*, and *CBL*, is less significant (*Φ*=0.023) than the mutations in the gene triple, *KIT*, *KRAS*, and *NRAS* (*Φ*=0.002). Interestingly, all the mutations in *JMJD1C* are germline variants (Wang *et al.* noted a significant enrichment of germline variants in *JMJD1C*). Thus, CoMEt identified mutual exclusivity between germline mutations in *JMJD1C* and somatic mutations in *CBL*.

We further investigated these modules by running CoMEt with parameters *α*=3,*t*=2,*k*=3 in order to to identify multiple gene sets simultaneously (Additional file 2: Table S9). The highest scoring collections included *KIT* and *KRAS* in one gene set, and *JMJD1C* and *CBL* in the other. This suggests that the mutations in these two pairs of genes co-occur, and indeed 6 samples have a mutation in either *JMJD1C* or *CBL* and either *KIT* or *KRAS* (co-occurrence *P*=0.28 by Fisher’s exact test). With only 53 samples in this dataset, it is difficult to identify all of the subtle relationships between mutual exclusivity and co-occurrence in larger sets of genes. Nevertheless, these results show the advantages of CoMEt analysis over pairwise tests of mutual exclusivity.

### Comparisons to other methods on real data

We compared CoMEt to the Multi-Dendrix [21] and mutex [24] algorithms on the TCGA GBM dataset from Leiserson *et al.* [21] and the TCGA AML [3] dataset. We did not compare on the TCGA BRCA or STAD datasets because CoMEt analyzes subtype-specific mutations, while Multi-Dendrix and mutex do not. We also provide a separate comparison to the muex algorithm [23] on GBM data in Additional file 1: Results, as muex does not identify multiple sets of alterations simultaneously. We ran Multi-Dendrix and mutex with default parameters, except that we set the maximum size of the mutex result groups to 4. We compared the modules output by CoMEt with the consensus output by Multi-Dendrix and the default output of mutex. We list the modules identified by Multi-Dendrix and mutex in Additional file 2: Tables S10 and S11.

**Glioblastoma multiforme (GBM)** We compared CoMEt’s results to the consensus modules reported by Leiserson *et al.* with Multi-Dendrix [21] on the TCGA GBM dataset [1] from Leiserson *et al.* (Additional file 2: Table S10(a)). Both CoMEt and Multi-Dendrix identify modules overlapping the Rb, p53, and PI(3)K signaling pathways. However, there are several key differences. First, CoMEt correctly places *CDKN2A* in a module with both the Rb and p53 signaling pathways, consistent with the figure in the TCGA GBM publication [1], while Multi-Dendrix does not. Second, in the module overlapping the PI(3)K pathway, CoMEt includes *NF1* and amplifications in *EGFR*, the latter alteration not analyzed in the Multi-Dendrix publication [21].

We performed two comparisons with mutex. First, we ran mutex without an input signaling network. Mutex reported a single connected component with 125 genes (Additional file 1: Figure S6 and Additional file 2: Table S11(a)). Although this component overlaps the four signaling pathways mentioned in the TCGA GBM paper [1], too many genes are included due to pairwise exclusivity with individual genes in the well-known signaling pathways. This makes it difficult to interpret the results. Next, we ran mutex with its default input signaling network to see whether limiting the search space would improve the mutex results (Additional file 1: Figure S7 and Additional file 2: Table S11(b)). Again, mutex reported a single connected component, this time with 16 genes. The component contains multiple mutually exclusive relationships also reported by CoMEt (for example, exclusivity between mutations in *CDK4*, *RB1*, and *CDKN2A*), but the CoMEt results are much easier to interpret because they include multiple modules. Even without the prior knowledge of protein interactions, the CoMEt results are arguably superior to those of mutex.

The comparison between CoMEt and mutex demonstrates several key advantages of our approach. First, although mutex’s results were indeed improved when using the signaling network, the massive differences between mutex’s results with and without the network indicates that mutex relies heavily on the network for prior knowledge. By not using prior knowledge, CoMEt can identify more novel combinations of mutations. Second, mutex’s reliance on the signaling network makes it more difficult for it to handle different types of aberrations compared to CoMEt. This is because when using a signaling network, aberrations must be mapped to single genes. But this is typically difficult for copy number aberrations that span a large region containing many genes. Mapping these aberrations to a signaling network is a difficult computational problem, and may obscure the underlying exclusivity between these copy number aberrations and other alterations. In contrast, CoMEt handles any types of aberrations as separate entries in the alteration matrix.

**Acute myeloid leukemia (AML)** We ran Multi-Dendrix and mutex on the TCGA AML dataset [3]. We did not run mutex with a signaling network because many of the alterations in the AML dataset are for groups of genes (for example, protein tyrosine phosphotases; see [3]). Multi-Dendrix reports a single consensus module that includes 19 genes (Additional file 1: Figure S8 and Additional file 2: Table S10(b)), and mutex identifies a connected component with 17 genes (Additional file 1: Figure S9 and Additional file 2: Table S11(c)). The size and complicated topology of these results make them difficult to interpret, especially compared to the CoMEt results, which include four different modules with 3 to 7 alterations each (Fig. 4). However, it is clear that while both Multi-Dendrix and mutex identify mutually exclusive mutations also identified by CoMEt (for example, mutual exclusivity between mutations in *PML*-*RARα*, *NPM1*, and *RUNX1*), they also miss key relationships (for example, exclusivity of mutations between *TET2*, *IDH2*, and the protein tyrosine phosphatase group).

### Robustness of CoMEt results on real data

**Bootstrapping** We used a bootstrapping approach to determine the robustness of the results from CoMEt. We sampled with replacement from the TCGA GBM dataset from Leiserson *et al.* [21] to generate resampled datasets. For each resampled dataset, we ran CoMEt and compared the output modules to the modules obtained on the whole dataset. We recorded the number of genes in common and the number of additional genes found by CoMEt in the resampled datasets (Additional file 1: Figure S10(a)). CoMEt recovered an average of 11 from the 17 genes in the modules from the whole dataset, and found an average of 8 additional genes. The genes in the most exclusive triples were recovered the most often (Additional file 1: Figure S11(a)): *CDKN2A*(D)-*TP53*-*MDM2*(A) (at least 68 *%* of datasets), *CDKN2A*(D)-*CDK4*(A)-*RB1* (84 *%*), and *PTEN*-*PTEN*(D)-*IDH1* (88 *%*).

**Downsampling** We also compared the CoMEt results on TCGA GBM dataset from Leiserson *et al.* [21] to the results obtained with only half the samples from this dataset. We created 25 datasets, each containing a random selection of 131 (50 %) of the samples. For each 50 % dataset, we ran CoMEt and compared the output modules to the modules obtained on the whole dataset. We recorded the number of genes in common and the number of additional genes found by CoMEt in the resampled datasets (Additional file 1: Figure S10(b)). Across the 25 datasets, CoMEt recovered an average of 11 from the 17 genes in the modules from the whole dataset, and found an average of 7 additional genes. We also computed how often each of the genes and relationships found by CoMEt on the whole dataset were found in the 50 % down-sampled datasets. CoMEt recovered the pairs in the *CDK4*-*RB1*-*CDKN2A* triple 84 % of the time, and the pairs in the *TP53*-*MDM2*-*CDKN2A* and the *PTEN*-*PTEN*(D)-*IDH1* triples 48 % of the time (Additional file 1: Figure S11(b)). This demonstrates that the results of CoMEt are fairly robust to changes in the number of samples. However, the well-known cancer pathways are found less frequently than in the bootstrapping results above, demonstrating that robust detection of mutual exclusivity does require a sufficient number of samples. Further theoretical analyses of the number of samples required to detect mutually exclusive sets are reported in [55].

## Conclusions

We introduce the CoMEt algorithm for identifying collections of mutually exclusive alterations in cancer *de novo*, that is, with no prior biological knowledge. CoMEt uses a novel statistical score for exclusive alterations that conditions on the frequency of each alteration and thus can detect exclusivity of rare mutations. CoMEt overcomes large computational challenges in computing the score using a new algorithm for contingency table analysis, and in optimizing the score in genome-scale data using the first Markov chain Monte Carlo (MCMC) algorithm for identifying collections of multiple sets of exclusive alterations.

We demonstrate that CoMEt is superior to earlier *de novo* methods — Dendrix [16], muex [23], Multi-Dendrix [21], and mutex [24] — on simulated and real data. We then apply CoMEt to large mutation datasets from multiple TCGA cancer types [1, 3, 4, 6]. On each dataset, CoMEt identifies significantly exclusive collections of alterations that overlap well-known cancer pathways and also implicates novel cancer genes. In addition, CoMEt illustrates subtle relationships between mutual exclusivity resulting from cancer subtypes and exclusivity resulting from pathways or protein interactions. These findings provide testable hypotheses for further downstream analysis or experimental validation.

The input to CoMEt is a matrix of binary alterations, and thus can be used to analyze a variety of alterations including point mutations and indels, copy number aberrations (amplifications and deletions) and complex rearrangements, splice-site mutations, gene fusions, and subtype annotations. CoMEt may be useful in the analysis of other types of alterations, such as germline variants.

Another application for CoMEt is pan-cancer analysis, such as the recently published TCGA study [5] and the upcoming ICGC Pan-Cancer Project. Since pan-cancer datasets have many cancer-type-specific alterations, CoMEt’s ability to simultaneously analyze type-specific and other types of exclusive alterations should prove useful for this analysis. Finally, we anticipate that the novel tail enumeration strategy used in CoMEt may be of broader interest, both for examining mutual exclusivity in other datasets, including non-biological data, as well as for adapting for other types of exact statistics.

## Materials and methods

### CoMEt algorithm

We consider a set of *malterations* measured in *n* samples. An *alteration* can be a variety of different genomic, transcriptomic, or epigenomic changes measured in a cancer sample; e.g. the somatic mutation of gene, a mutation in a particular amino acid residue (such as the V600E mutations in the *BRAF* gene that are common in colorectal and other cancers [56]), or an epigenetic change such as hypermethylation of a promoter. We assume that alterations are binary: in each sample, an alteration either occurs or does not occur. We represent the status of *m* measured alterations in *n* samples with an *m*×*n* binary alteration matrix *A*=[*a*_*ij*_], where *a*_*ij*_=1 if alteration *i* occurs in sample *j*, and *a*_*ij*_=0 otherwise. We define a set of *k* measured alterations as an *n*×*k* submatrix *M*. Our goal is to identify a collection **M**=(*M*_1_,*M*_2_,…,*M*_*t*_) of *one or more* sets of mutually exclusive alterations across the *n* samples. We introduce the Combinations of Mutually Exclusive Alterations (CoMEt) algorithm for this purpose (see Fig. 2).

**Scoring mutual exclusivity** CoMEt uses a novel statistical score based on an exact test for mutual exclusivity. Figure 1 motivates the development of the new score, showing two sets *M* and *M*^′^, each with four alterations. The alterations in both sets are perfectly exclusive (no sample has more than one alteration), and the total number of altered samples is the same. The Dendrix weight function *W*(*M*) introduced in [16] (and used in later publications [19–21]) is defined as the *coverage*, the number of samples with at least one mutation in *M*, minus the *coverage overlap*, the number of samples with more than one mutation in *M*. In this case, *W*(*M*)=*W*(*M*^′^). However, given the frequencies of each alteration, we are more surprised to observe mutual exclusivity among alterations in the set *M*^′^, which are each altered in 7 *%* of samples, than we are to observe mutual exclusivity among the alterations in set *M*, where a single alteration has very high frequency (25 %) and three alterations have relatively low frequency (<2 *%*). Sets like *M* are common in many cancer datasets where highly recurrent alterations (such as mutations in *TP53* or amplification of *EGFR*) occur and can be combined with low frequency, spurious alterations.

We first describe a statistical score *Φ*(*M*) for a tuple *M*=(*m*_1_,…,*m*_*k*_) of alterations. The score measures the surprise of the observed exclusivity of these alterations *conditional* on the rate of occurrence of each alteration. Since these rates are generally unknown (for example, the background mutation rate for single nucleotide mutations varies greatly across genes and samples [57]), we use the *exact distribution* obtained from the observed data as the null distribution. Under this distribution, the status of the *k* alterations in *n* samples is described by selecting uniformly a *k*×*m* binary alteration matrix *B* with the constraint that the number of 1’s in row *i* of *B* equals the number of 1’s in row *m*_*i*_ of the alteration matrix *A*. This distribution is equivalent to the sampling distribution on 2×2×⋯×2=2^*k*^ contingency tables under the hypergeometric distribution, where dimension *i* of the table gives the cross-classification of the number of samples where alteration *i* occurs or not. For example, three alterations are described by a 2×2×2 table with margins equal to the frequency of each alteration (Fig. 1(b)).

We introduce notation to describe the statistical test. Given a set *M* of alterations, let $x^{+}_{(j)}$ be the number of samples where alteration *m*_*j*_ occurs. It follows that $n - x^{+}_{(j)}$ is the number of samples where *m*_*j*_ does not occur. Similarly, for **v**⊆ [ *k*]={1,…,*k*}, let *x*_**v**_ denote the number of samples where alterations only occur in *m*_**v**_. The values *x*_**v**_ for all *v*⊆ [ *k*] give the entries of a 2^*k*^ contingency table **X**_*M*_ with fixed margins $\mathbf {x^{+}} = \left (x^{+}_{(1)}, \dots, x^{+}_{(k)}\right)$. Thus, the probability of observing a 2^*k*^ contingency table **X**_*M*_ with fixed margins *x*^+^ and whose sum of entries equals *n* follows the multivariate hypergeometric distribution

(2) $$  p_{\mathbf{X}_{M}} = \Pr(\mathbf{X}_{M}| \mathbf{x^{+}}, k, n) = \frac{\prod_{j=1}^{k} x^{+}_{(j)}! \left(n -x^{+}_{(j)} \right)! }{ (n!)^{k-1} \prod_{\mathbf{v} \subseteq [k]} x_{\mathbf{v}}!}.  $$

To characterize the mutual exclusivity of alterations in a contingency table, we define the test statistic as the sum of the entries in the contingency table where *exactly* one alteration occurs, that is, $T(\mathbf {X}_{M}) =\sum _{j=1}^{k} x_{\{j\}}$, where *x*_{*j*}_ is the number of samples where alterations occur only in *m*_*j*_. We compute a *P*-value for the observed value *T*(**X**_*M*_) of the test statistic as the tail probability of observing tables with the same margins whose exclusivity is at least as large as observed:

(3) $$  \Pr\left(T \ge T(\mathbf{X}_{M}) | \mathbf{x^{+}}, k, n \right) = \sum_{\substack{\mathbf{Y} \in \mathcal{T}(\mathbf{x^{+}}): \\ T(\mathbf{Y}) \ge T(\mathbf{X}_{M})}} \Pr\left(\mathbf{Y} | \mathbf{x^{+}}, k, n \right),  $$

where $\mathcal {T}(\mathbf {x^{+}})$ is the set of 2^*k*^ contingency tables with margins **x**^+^. Note that for *k*=2, the test statistic *T*(**X**_*M*_) is equivalent to a one-sided Fisher’s exact test. 2×2 contingency tables have only one degree of freedom, and thus there are essentially only two ways in which the corresponding pair of random variables can be non-independent: having too many co-occurrences or too much exclusivity (Fig. 1(b)). However, 2^*k*^ tables have 2^*k*^−*k*−1 degrees of freedom and there are many ways in which the corresponding random variables can be non-independent. The *T*(**X**_*M*_) test statistic measures whether the alterations are surprisingly *mutually exclusive*, rather than non-independent in some other way.

We define the score *Φ*(*M*) using the mid *P*-value [58], which is the the average of the probability of observing a value at least as extreme as the observed value and observing a value more extreme than observed:

(4) $$\begin{array}{*{20}l} \Phi(M) = \quad \frac{1}{2} (Pr(T \ge T(\mathbf{X}_{M}) | \mathbf{x^{+}}, k, n) \\  \quad + Pr(T > T(\mathbf{X}_{M}) | \mathbf{x^{+}}, k, n)). \end{array} $$

We use the mid *P*-value because the tail probability from exact tests is typically overly conservative, due to the discreteness of the exact distribution [58]. Finally, since cancer is driven by mutations in multiple pathways [15], we define a score *Φ*(**M**) for a collection **M**=(*M*_1_,*M*_2_,…,*M*_*t*_) of *t* gene sets as $\Phi (\mathbf {M}) = \prod _{i=1}^{t} \Phi (M_{i})$. The product results from our assumption that under the null hypothesis mutations in different sets *M*_*i*_ are indep endent.

### Computing the mutual exclusivity score *Φ*(*M*)

To compute the mutual exclusivity score *Φ*(*M*), one must compute (3). This requires computing the probability of all tables **Y** with the same margins as **X**_*M*_ and with exclusivity statistic *T*(**Y**) at least as large as the observed value *T*(**X**_*M*_). Unfortunately, no algorithm is known to enumerate such tables. In general the problem of counting contingency tables with fixed margins is #P-complete [59], and thus it is unlikely they can be enumerated efficiently. Several methods have been proposed to solve the problem of counting contingency tables, including using the network algorithm [60, 61] for Fisher’s exact test in *r*×*c* contingency tables, or extensions to consider the joint effect of two contingency tables (that is, 2×*r*×*c*) [62]. Branch and bound heuristics have also been used in some specialized cases [63]. However, these approaches still consider at most three-dimensional contingency tables, and the problem of enumerating 2^*k*^ tables does not seem to have been considered. Even for small *k* the enumeration problem is intractable: the number of 2^*k*^ tables with fixed margins grows exponentially in *k*. The work [64] presented an exhaustive algorithm to enumerate all 2^3^ and 2^4^ contingency tables with fixed margins, demonstrating for example that for *n*=36, there are >100 million 2^4^ tables. Randomized and approximate counting methods for contingency tables have been developed (see, for example, [65, 66] and references therein), although these generally do not provide a rigorous guarantee on the error in the approximation.

We derive a novel *tail enumeration* algorithm to efficiently compute the tail probability in Eq. (3) for tables with high values of the exclusivity statistic *T*. The motivation for our approach is that the sets *M* of interest will have extremely high values of *T*(**X**_*M*_), near the maximum possible value. For example, in the degenerate case of perfect exclusivity (no sample with more than one alteration in *M*) there are no more extreme tables to enumerate, and the algorithm needs only to evaluate the hypergeometric probability of Eq. (2) for this single table. Thus, if we enumerate tables starting from the highest possible values for *T*, we can obtain highly accurate *P*-values for the most interesting cases. Furthermore, we can stop the enumeration procedure when the *P*-value becomes sufficiently large and use approximations for these larger *P*-values (see below).

Algorithm 1 is the tail enumeration strategy to enumerate contingency tables in approximate order from most to least exclusive. Briefly, let **C**=(**v**⊆ [ *k*]:|**v**|≥2) be the vector of co-occurring (not exclusive) cells. The basic strategy employed by Algorithm 1 is to generate a table **Y** that is more exclusive than **X**_*M*_ (that is, *T*(**Y**)>*T*(**X**_*M*_) by iterating through the possible values of each cell in **C**, using the following facts:

- When all values in **C** are fixed, the other values in the contingency table are uniquely determined (see Procedure CompleteContTbl in Algorithm 1).
- We can set and update exact upper and lower bounds for each cell in **C**. The values of each cell are bounded by two values (lines 10–11 in TailEnumeration): the first is how many more co-occurrences are allowed in the current table (*T*_*REM*_) before **Y** is less exclusive than **X**_*M*_; the second is given by the constrained marginal (*MarRem*) for that variable in **X**_*M*_.

We find that Algorithm 1 performs well on real data, evaluating the test statistic *T*(**X**_*M*_) in a few seconds for sets with *k*≤7 that have a small number of co-occurrences.



**Binomial approximation.** We can approximate the distribution of the exclusivity statistic using the binomial distribution, which is a well-known approximation of the hypergeometric distribution. Under the null hypothesis that alterations occur independently in the samples, let $p_{e} = \sum _{j=1}^{k} \frac {x_{(j)}}{n}$ be the probability of an exclusive alteration; that is, a sample contains exactly one alteration in *M*. Given a set *M* of alterations *M*, then the probability of observing *T*(**X**_*M*_) or more exclusive alterations in *n* samples is given by the binomial tail probability $1 - \sum _{i=0}^{T(\mathbf {X})-1} {n \choose i} {p_{e}^{i}} (1-p_{e})^{n-i}$.

We find that the binomial provides a good approximation of the exact test *P*-value for sets *M* with a large number of co-occurring mutations, and consequently a higher *P*-value (see Additional file 1: Figure S12). Conveniently, these are precisely the cases where the tail enumeration algorithm is slow.

**Permutation approximation.** Another approximation to the exact test is obtained using a permutation test. We sample *L* tables with fixed margins uniformly from the space of all tables and compute the proportion of such tables whose exclusivity value *T* exceeds the observed value *T*(**X**_*M*_). Of course, sampling uniformly from the set of tables with fixed margins is not straightforward. We use an MCMC approach as described in [18], although we do not fix the number of alterations per sample. Interestingly, while the MEMo algorithm [18] uses a permutation test, the test statistic is the coverage *Γ*(*M*), rather than the exclusivity *T*(*M*) used in CoMEt. While these are equivalent when *k*=2 (since there is only one degree of freedom), they produce different results for *k*>2. See further discussion in the section Comparison to MEMo.

In our implementation, we use the exact test, binomial approximation, or permutation approximation to compute *Φ*(*M*) according to the following procedure. First, we calculate the *P*-value from the binomial approximation and compute the number of co-occurring alterations in *M*. If the number of co-occurring alterations is higher than a fixed threshold *κ* or the binomial *P*-value is larger than a fixed value *ψ*, we set *Φ*(*M*) to be the binomial *P*-value. Otherwise, we perform the tail enumeration procedure to compute the exact test *P*-value, stopping the enumeration if the accumulated tail probability becomes larger than a threshold *ε*. If we stop, then we compute the permutation approximation with $\lceil {\frac {1}{\epsilon }}\rceil $ samples, such that we expect to sample at least one table with *T*>*T*(**X**_*M*_). This procedure focuses the time to perform tail enumeration in those cases where high accuracy is needed for small *P*-values.

### Sampling collections of mutually exclusive alterations with MCMC

Our goal is to identify a collection **M** of *t* alteration sets with low (highly significant) values of *Φ*(**M**). Since it is typically not possible to enumerate all such collections (except for test datasets with small *m*, *n*, *t*, and *k*), we derive a Markov Chain Monte Carlo (MCMC) approach to sample from the space of possible collections. We use the Metropolis-Hastings algorithm [67, 68] to derive an MCMC algorithm to sample collections **M** in proportion to the weight *Φ*(**M**)^−*α*^, where higher values of *α* increase the sampling frequency of the most mutually exclusive sets (see Additional file 1: Section Methods for additional details). We use *α*=2 except where noted.

#### Choosing values for *t* and *k*

Ideally, CoMEt should be run with the largest values of *k* and *t* that are biologically meaningful for a particular dataset. If smaller values of *k* and *t* are best supported by the data, the summarization procedure will demonstrate this. We see examples of this in glioblastoma, where the ten most significant collections identified by CoMEt include a set with *TP53*, *MDM2*, *MDM4*, and one of five other alterations (Additional file 2: Table S5).

In practice, using large values of *k* and *t* might lead to long run times and slow convergence of the MCMC algorithm, since the space of possible collections will be very large. Thus, an alternative approach that we use to generate results is to run with small values of *t* and *k* (for example, *t*=3,4 and *k*=3,4) and examine the resulting marginal probability graph. If there are *t* or more cliques or approximate cliques in the graph, this suggests the use of larger values of *t* and *k*. We used this approach to find larger collections in the AML dataset (see details in section CoMEt results on real cancer datasets).

### Marginal probability graph

We now present a method to extract a collection of highly exclusive alteration sets (*with no prescribed size*) from the posterior distribution obtained from the MCMC algorithm. Typically, there are multiple collections with significant scores. This might occur for interesting reasons such as different sets of alterations with similar scores or alterations that appear in multiple mutually exclusive sets. However, the reason might also be suboptimal parameter selection; for example, there may be a significant set of *k*=3 alterations in the data, but running the algorithm with *k*=4 will return many sets with the same three genes and a fourth “spurious” gene. To distinguish such cases, we summarize the posterior distribution on collections using a *marginal probability graphG*. For a pair (*i*,*j*) of alterations, let *p*(*i*,*j*) denote the posterior probability that *i* and *j* are found in the same set. We compute *p*(*i*,*j*) using the samples from the MCMC algorithm (see Additional file 1: Section Methods).

Let *G*=(*V*,*E*) be a complete, undirected weighted graph whose vertices are the alterations and where each edge *e*∈*E* connects a pair of vertices *u*,*v* with weight *p*(*u*,*v*). Connected subgraphs of *G* with many high-weight edges are the most exclusive alteration sets in *A*. We identify these most exclusive alteration sets by first removing all edges with weight below a threshold *δ* (see Additional file 1: Section Methods). Let *C*(*δ*) be the connected components of size ≥2 in the resulting graph. The output of CoMEt is the *C*(*δ*) alteration sets. We choose connected components as the output — as opposed to some other partition of the graph such as cliques — in order to be able to identify other topologies such as overlapping pathways (alteration sets), where two sets of alterations are connected by a cut node.

### Statistical significance

While the score *Φ*(**M**) measures our surprise of observing exclusivity within each of the sets in **M** conditional on the observed frequencies of each alteration, there is a large number of possible collections, and thus we might observe a high score by chance. We evaluate the statistical significance of the collection **M** by comparing to a null distribution of scores obtained on permuted alteration matrices *A* with the sample and alteration frequencies (sums of rows and columns of *A*) fixed [18, 69]. Let *Φ*^∗^ be the minimum score obtained over *N* permutations. We use the collections **M** satisfying *Φ*(**M**)≤*Φ*^∗^ (thus each such collection has *P*-value $ < \frac {1}{N}$) to compute the marginal probability graph except where noted.

### Simultaneous analysis of alterations and cancer subtypes

An important confounding factor in identifying cancer pathways *de novo* by analyzing exclusive alterations is that certain alterations primarily occur in particular cancer subtypes [38]. If we analyze a mixed set of samples with multiple subtypes, these subtype-specific alterations will be mutually exclusive in the data, even if they are not in the same biological pathway. When the subtypes are known in advance, one solution is to analyze subtypes separately; unfortunately, this reduces sample numbers, thus reducing power to identify combinations of alterations that are shared across subtypes. CoMEt addresses this problem by adding one new “subtype row” to the alteration matrix *A* for each subtype. This subtype row contains an alteration in all samples *excluding* those of the given subtype. Thus, the sets of alterations that are surprisingly exclusive with these subtype rows are the ones primarily *altered* in that subtype. Note that when running CoMEt with subtype rows, we do not allow multiple subtypes to be placed in the same set. Because CoMEt simultaneously analyzes multiple alteration sets, it can identify exclusive sets containing subtype-specific alterations, general alterations, or any combination of these.

When analyzing the cancer dataset that included sample subtype classifications, we perform two runs of CoMEt. First we run CoMEt on the alteration matrix *A*. Then we run CoMEt on the alteration matrix with “subtype rows” as we described. We summarize the ensemble of statistically significant collections sampled by the MCMC algorithm in the two CoMEt runs by normalizing and combining the sampling frequencies of each collection across the two runs, and then computing the marginal probability graph on the merged collection.

### Somatic mutation datasets

**Acute myeloid leukemia (AML)** The AML dataset contains whole-exome and copy number array data in 200 AML patients from The Cancer Genome Atlas (TCGA) [3]. Using the annotations in [3], we have categorized multiple genes together based on expert knowledge, which results in 9 categories including spliceosome, cohesin complex, MLL-X fusions, other myeloid transcription factors, other epigenetic modifiers, other tyrosine kinase, serine/threonine kinase, protein tyrosine phosphatase, and RAS protein. More details are given in [3]. This results in 51 genes and 200 patients.

**Glioblastoma multiforme (GBM)** We analyzed three GBM datasets:

1. TCGA GBM dataset from Leiserson *et al.* [21]. This dataset contains whole-exome and copy number array data in 261 GBM patients and 398 genes from TCGA [1]. Data preparation for GBM can be found in [21]. Note that in section Glioblastoma multiforme (GBM) we included amplifications in *EGFR* which were not considered in [21]. Also, we mapped deletions in *FAF1* to *CDKN2C*, since these genes are adjacent on chromosome 1, and *CDKN2C* is the likely target of the aberration.
2. TCGA GBM dataset from Szczurek *et al.* [23]. This dataset contains 83 alterations in 236 samples from [1], including single nucleotide variants in genes identified as significantly mutated by MutSigCV [9] and copy number aberrations caelled by GISTIC2 [70] then restricted to those with significantly concordant gene expression (higher for amplifications, lower for deletions).
3. TCGA GBM dataset from the TCGA Pan-Cancer project [5]. We analyzed the non-silent mutations (single nucleotide variants and small indels) from the mutation annotation format (MAF) file and focal copy number aberrations from GISTIC2 output. This dataset contains 509 genes in 291 samples. Moreover, we removed genes with non-silent mutations in <1 *%* of samples and with mutations in >2.5 *%* of samples with MutSigCV [9] *q*-value >0.1. This dataset contains 406 genes in 291 samples.

**Gastric cancer (STAD)** We analyzed the non-silent mutations (single nucleotide variants and small indels) from the MAF file in 289 gastric cancer samples. We also included focal driver copy number aberrations from GISTIC2 output via Firehose, fusion genes, rearrangements and splicing events [6]. We removed 74 hypermutators and genes with non-silent mutations in <2.5 *%* of samples and with mutations in >3 *%* of samples with MutSigCV [9] *q*-value >0.25. This process results in 217 STAD patients and 397 genes with mutations. We considered four subtypes identified by TCGA [6], including tumors positive for the Epstein-Barr virus (EBV), tumors with high microsatellite instability (MSI), genomically stable (GS) tumors with a low level of somatic copy number aberrations, and chromosomally unstable (CIN) tumors with a high level of somatic copy number aberrations that were called. We do not analyze the MSI subtype since samples in MSI are hypermutated.

**Breast cancer (BRCA)** The BRCA dataset contains whole-exome and copy number array data in 507 BRCA patients and 375 genes from TCGA [4]. Data preparation for BRCA can be found in [21]. We downloaded subtype information of BRCA from TCGA [4]. We considered four subtypes — basal-like, HER2-enriched, luminal A, and luminal B — that each contain at least 10 *%* of the total samples.

We list the barcodes of the TCGA samples in each of the datasets in Additional file 2: Table S12.

### Comparison to MEMo

The MEMo algorithm [18] uses a permutation test to approximate the probability of observing exclusive mutations in a gene set *M* with contingency table *X*. The permutation test works by permuting the rows in *A* corresponding to the genes in *M*, and then determining if the permutation has a higher test statistic than *M*. This is then repeated *N* times to obtain an empirical *P*-value.

The crucial difference between MEMo and CoMEt is that MEMo uses the coverage *Γ*(*M*) as the test statistic, while CoMEt uses the test statistic *T*(*X*). (For ease of exposition, let *Γ*(*X*) also be defined as the coverage for a contingency table *X*). The reasoning behind using the coverage as the test statistic is the idea that a gene set with mutually exclusive alterations will also have the highest coverage possible, for fixed frequencies of individual alterations. While this is true for pairs of genes (which follows from the fact that 2×2 contingency tables have only one degree of freedom), when one examines three or more genes, maximizing coverage is not the same as maximizing exclusivity. In fact, we can see that for a given contingency table *X* it is possible to find another contingency table *X*^′^ with the same margins (gene frequencies) as *X*, but that has:

1. Higher exclusivity (*T*(*X*^′^)>*T*(*X*)) and lower coverage (*Γ*(*X*^′^)<*Γ*(*X*)), which could result in a deflated *P*-value for MEMo.
2. Lower exclusivity (*T*(*X*^′^)<*T*(*X*)) but the same coverage (*Γ*(*X*^′^)=*Γ*(*X*)), which would result in an inflated *P*-value for MEMo.^2^

See examples of both cases in Additional file 1: Figure S13.

### Comparison of CoMEt and mutex methods

The recently introduced mutex [24] algorithm uses an iterative version of the one-sided Fisher’s exact test to evaluate combinations of mutually exclusive alterations. Thus, the tests used in mutex and CoMEt are identical when evaluating the exclusivity of a pair of alterations. However, for *k*>2 alterations, mutex and CoMEt are quite different. CoMEt directly assesses the exclusivity of a 2^*k*^ contingency table. In contrast, mutex computes a series of 2×2 tests examining the exclusivity of alterations in one gene compared to the alterations in all *k*−1 other genes in the set. For a set with *k*>2 genes, mutex returns the least significant (highest) *P*-value of these 2×2 tests. While mutex’s method is faster to compute than CoMEt, it is not as powerful at detecting mutual exclusivity in sets of *k*>2 alterations, as shown in in section Benchmarking of methods for individual gene sets. Furthermore, while mutex searches for sets with *k*>2 genes using a greedy approach to gradually expand mutually exclusive pairs, CoMEt uses an MCMC algorithm to simultaneously sample a collection of mutually exclusive sets. Searching for multiple sets simultaneously was shown to have advantages over the greedy approach in [21]. Finally, CoMEt summarizes the posterior distribution of the significant collections. Typically, CoMEt output contains multiple distinct modules. In contrast, mutex tends to produce results with many more genes, requiring prior knowledge in the form of an interaction network to reduce the search space.

### Comparison of methods with and without mutation filtering

Because CoMEt conditions on the observed alteration frequencies, we argue that it is less biased towards genes that have high frequencies of passenger mutations, such as long genes. To illustrate this point, we compared CoMEt, Multi-Dendrix, and mutex on glioblastoma (GBM) data with and without the MutSigCV [9] filter that requires that frequently mutated genes have low MutSigCV *q*-values (see section Somatic mutation datasets for details). We ran CoMEt with *k*=4 and *t*=4, ran Multi-Dendrix with its default parameters of *t* ranging from 2 to 4 and *k* ranging from 3 to 5, and ran mutex with default parameters except that we set the maximum size of a result group to 4 and did not include a signaling network. We used mutation data from the TCGA Pan-Cancer dataset [5] which contains whole-exome and copy number array data, and downloaded MutSigCV output from the corresponding Synapse repository (syn2812925). We used different TCGA GBM datasets here than in section Glioblastoma multiforme (GBM) because of the availability of MutSigCV results on the Pan-Cancer dataset. For each cancer, we generated two datasets. In one dataset, we applied a MutSigCV filter to remove highly altered genes (altered in >2.5 *%* of samples) but insignificant by MutSigCV (*q*-value <0.1). The second dataset did not include any MutSigCV filter.

We found that CoMEt identifies the key combinations of mutated genes with or without the mutation filtering on the GBM dataset (Table 1 and Additional file 1: Figure S14(a)). These key combinations include genes from the Rb signaling (*CDK4*, *RB1*, *CDKN2A*), p53 signaling (*TP53*, *MDM2*), and PI(3)K signaling (*PIK3CA*, *PTEN*, *IDH1*) pathways, as well as *EGFR* and *NF1*. The CoMEt results were also largely stable: the core members of each module were unchanged, while four genes with less clear roles in GBM were lost and four genes were gained when we removed the mutation filtering.

**Table 1:** Comparison of CoMEt, Multi-Dendrix, and mutex on the TCGA GBM dataset from the TCGA Pan-Cancer project [5] with and without mutation filtering. The consensus modules output by each algorithm are shown for the dataset with and without mutation filtering. The (A) and (D) following the gene names indicate amplifications and deletions, respectively

Algorithm	Without filtering	With filtering
CoMEt		
	1. *IDH1, PIK3CA, PTEN,* ***KSR2***	1. *IDH1, PIK3CA, PTEN,* ***DNAH11***
	2. *MDM2*(A), *RPL5, STAG2, TP53*	2. *MDM2*(A), *RPL5, STAG2, TP53,* ***SEMA3E***
	3. *EGFR, NF1,* ***CALCR, PCDHA3, PPP1R3A***	3. *EGFR, NF1,* ***PKHD1, THSD7B***
	4. *CDK4*(A), *CDKN2A*(D), *PTPN11, RB1, ZNF407*	4. *CDK4*(A), *CDKN2A*(D), *PTPN11, RB1, ZNF407*
Multi-Dendrix		
	1. *CNTNAP2, CDKN2A*(D), *CDK4*(A), *EGFR, IDH1, MDM2*(A), *MDM4*(A), *NF1, PIK3CA, PTEN, RB1,* ***COL6A3, MAST4, PCDHA3, PCLO, PDGFRA*** **(A)**, ***PIK3R1***	1. *CNTNAP2, EGFR, IDH1, MDM2*(A), *MDM4*(A), *PTEN, TP53,* ***ATRX, CHD9, HRNR, MUC4, MUC16, TTN***
		2. *CDKN2A*(D), *CDK4*(A), *NF1, RB1,* ***FRG1B***
mutex	1. *CDK4*(A), *CDKN2A*(D), *EHD3, MAST4, NF1, PTPN11, RB1*	1. *CDK4*(A), *CDKN2A*(D), *EHD3, MAST4, MDM2*(A), *NF1, PTPN11, RB1, STAG2, TP53,* ***CACNA1S, CALCR, DGKD, EGFR, FRG1B, PKHD1, THSD7B, ZNF407***
	2. *MDM2*(A), *STAG2, TP53*	

Bolded genes indicate differences in output with and without mutation filtering

In contrast, Multi-Dendrix and mutex results change more substantially, with and without mutation filtering. The Multi-Dendrix modules are shuffled considerably, including the group of key GBM cancer genes (Table 1 and Additional file 1: Figure S14(b)). In addition, six genes are lost and seven genes are gained after we remove mutation filtering. Furthermore, many of the genes that are added without mutation filtering are known to have elevated mutation rates, including *TTN*, *MUC16*, and *MUC4* [9]. This demonstrates a deficiency of the Dendrix weight function, also used by Multi-Dendrix, in that high coverage (frequently altered genes) may dominate over mutual exclusivity. The modules output by mutex also change considerably with and without mutation filtering (Table 1 and Additional file 1: Figure S14(c)). Without mutation filtering, the number and composition of each module change, and eight genes are added. Moreover, mutex did not report the strong exclusive set from the PI(3)K signaling pathway (*PTEN*, *PIK3CA*, *IDH1*) found by CoMEt.

## Endnotes

^1^ We note that while Multi-Dendrix [21] and mutex [24] can identify overlapping gene sets, this feature was not explored in the corresponding publications.

^2^ We have not found a case where *T*(*X*^′^)<*T*(*X*) and *Γ*(*X*^′^)>*Γ*(*X*), and conjecture that such a case does not exist.

## Additional files

Additional file 1
**Supplementary information.** Supplementary results and figures. (PDF 5440 kb)

Additional file 2
**Supplementary tables.** (XLSX 350 kb)
