# Erratum to: CoMEt: a statistical approach to identify combinations of mutually exclusive alterations in cancer

**DOI:** 10.1186/s13059-016-1034-9

**Published:** 2016-08-02

**Authors:** Mark D. M. Leiserson, Hsin-Ta Wu, Fabio Vandin, Benjamin J. Raphael

**Affiliations:** 1 Department of Computer Science, Brown University, 115 Waterman Street, Providence, RI 02912 USA; 2 Center for Computational Molecular Biology, Brown University, Box 1910, Providence, RI 02912 USA; 3 Department of Mathematics and Computer Science, University of Southern Denmark, Campusvej 55, Odense M, Denmark

## Erratum

After the publication of this work [1] it has been brought to our attention that the descriptions of the generation of the two simulated datasets were confusing.

In the section ‘Benchmarking of methods for individual gene sets’, the first sentence of the second paragraph should specify the number of genes included in the gene set as 100, and it should read:

“We compared CoMEt to the other methods on datasets with *m* = 100 genes and *n* = 500 samples and with implanted pathways with coverages *γ* ranging from 0.1 to 1.0.”

In the section ‘Benchmarking identification of collections of gene sets’, the first paragraph should specify that genes mutated in fewer than 1% of total samples (that is in fewer than 5 out of 500 samples) were removed from the simulation, and the sixth sentence in this paragraph should read:

“Third, we include *m* = 20,000 genes and remove those genes that are mutated in fewer than 1% of total samples (that is in fewer than 5 out of 500 samples) (Additional file 1: Figure S2).”

Additionally, these descriptions are also included in the Additional file 1, section S3, and should read:

“We generated two different versions of the simulated datasets, depending on whether we implanted a single or multiple gene sets. We describe the method for generating datasets with multiple implanted gene sets in the main text. For all simulated datasets, we used n = 500, |C| = 5, *γ*_*C*_ = (0.67, 0.49, 0.29, 0.29, 0.2), and q = 0.0027538462.1 We used *μ*_*P*_ = (0.5, 0.35, 0.15) for the single pathway simulations.”

We also updated the description of our procedure for assessing the convergence of the MCMC algorithm in Additional file 1, section S2, which should read:

“To assess the convergence of the MCMC algorithm, we ran multiple chains with different initializations. For one of these initializations, we used the collection output by Multi-Dendrix [21] (using the same values of the parameters t and k as in CoMEt). The remaining initializations were random collections. We ran the MCMC algorithm with these initializations, running each chain for a given number of iterations. We consider the chains converged if the mean total variation distance between the chains is smaller than 0.005. Otherwise, we increase the number of iterations by a factor of 1.5. We repeat this process until the chains converge or the total number of iterations per chain reaches a maximum number of iterations, which we set as 1 billion. The output of the MCMC algorithm is the union of the sampling distributions from the different initializations.”

The corrected Additional file 1 is included in this Erratum.

## Additional file

Additional file 1: Supplementary information. Supplementary results and figures. (PDF 5528 kb)
