# Supplementary material for: Erratum to: CoMEt: a statistical approach to identify combinations of mutually exclusive alterations in cancer
Source: Genome Biol. 2016 Aug 2;17:168. doi: 10.1186/s13059-016-1034-9 (PMC4970302; doi:10.1186/s13059-016-1034-9)
Supplement: Additional file 1: — Supplementary information. Supplementary results and figures. (PDF 5528 kb) [file 13059_2016_1034_MOESM1_ESM.pdf]

Supplementary Information for

CoMEt: A Statistical Approach to Identify Combinations of Mutually Exclusive Alterations in Cancer

Mark D.M. Leiserson[1,2,*], Hsin-Ta Wu[1,2,*], Fabio Vandin[1,2,3], Benjamin J. Raphael[1,2]

[1]*Department of Computer Science and* [2]*Center for Computational Molecular Biology, Brown University, Providence, RI, USA*
[3]*Department of Mathematics and Computer Science, University of Southern Denmark, Odense M, Denmark*
[*]*Equal contribution.*

Correspondence: `braphael@brown.edu`

S1 Results

Comparison to muex on real data

We compared CoMEt to muex [23] using two different versions of the TCGA glioblastoma (GBM) dataset: (1) the dataset from Leiserson *et al.* [21] containing 398 alterations and 261 samples; (2) the dataset from Szczurek *et al.* [23], containing 83 alterations and 236 samples (See Section § *Glioblastoma multiforme (GBM)*). There are 184 samples in both the Multi-Dendrix GBM and muex GBM datasets. Besides the samples, the main difference between these two datasets is that the muex dataset is restricted to only 83 significantly recurrent alterations.

Since the muex score is for single alteration sets, we ran muex iteratively to identify collections of alteration sets. That is, we run muex to find the top scoring alteration set, remove those alterations, and repeat $t-1$ times. We ran muex with the parameters used in [23], restricting to alteration sets with coverage at least 0.3, impurity lower than 0.5, and a significance cutoff of 0.05. On the muex GBM dataset, we ran CoMEt and muex with $k=4$ and $t=3$ to match the parameters used in [23]. On the Multi-Dendrix GBM dataset, we ran CoMEt and muex with $k=3$ and $t=3$, since muex aborted with an out-of-memory error for $k=4$ on this dataset.

On both GBM datasets, CoMEt identifies collections with much more significant exclusivity. Moreover, more of the genes in the CoMEt collections are known cancer genes (according to the COSMIC Cancer Census [71]) compared to the genes in the muex collections (Table S13). On the Multi-Dendrix GBM dataset, CoMEt identifies three collections that overlap the Rb (*CDK4*, *CDKN2A*, *RB1*), p53 (*TP53*, *MDM2*, *CDKN2A*), and PI(3)K (*PTEN*, *IDH1*) signaling pathways. Each of these sets include surprisingly exclusive alterations, with $\Phi(M)$ ranging from 10^{-8} to 10^{-19}, and all the alterations are in cancer genes. In contrast, muex identifies sets with lower coverage and less surprising exclusivity, with $\Phi(M) > 10^{-3}$ for each set, and three of the alterations are not in known cancer genes.

On the muex GBM dataset, CoMEt again identifies more exclusive alteration sets that overlap more known cancer genes, while muex reports few known cancer genes with most having an uncertain association with cancer. In general this dataset seems to include more spurious alterations, as both algorithms identify less exclusive sets with fewer cancer genes than on the Multi-Dendrix GBM dataset. This might be a result of the different handling of copy number aberrations in the two papers (see [21] and [23]).

S2 Methods

MCMC Algorithm

We define a Markov chain whose states Ω are possible collections $\mathbf{M}$ and where transitions between states (collections) are defined such that the chain is ergodic. Finite and ergodic Markov chains converge to a unique stationary distribution. In this case, because we want to sample from collections $\mathbf{M}$ in proportion to their weights

$$\Phi(\mathbf{M})^{-\alpha} = \prod_{M \in \mathbf{M}} \Phi(M)^{-\alpha},$$

our desired stationary distribution is

$$\pi_{\mathbf{M}} = \frac{\Phi(\mathbf{M})^{-\alpha}}{\sum_{\mathbf{M}' \in \Omega} \Phi(\mathbf{M}')^{-\alpha}}. \quad (1)$$

Note that we use $\Phi(\mathbf{M})^{-\alpha}$ so more exclusive collections have higher weights. The Metropolis-Hastings algorithm [67,68] is a method for defining transition probabilities for an irreducible Markov chain such that the modified chain is ergodic and has a desired stationary distribution. A Metropolis-Hastings algorithm to sample collections $\mathbf{M}$ according to this stationary distribution is as follows:

Initialization. Choose tk genes uniformly at random from $\mathcal{E}$, and assign k genes at random to initialize $\mathbf{M} = M_1, \ldots, M_t$.

Iteration. For $N = 1, 2, \ldots$, obtain $\mathbf{M}_{N+1}$ from $\mathbf{M}_N$ as follows:

1. Select a gene g uniformly at random from $\mathcal{E}$.
2. Define the proposed collection $\mathbf{M}'_N$ as follows:
   i) If $g \notin \mathbf{M}_N$, then choose uniformly at random gene $g' \in M_i$, and replace g' with g.
   ii) Else, choose uniformly at random gene $g' \in M_i$, and *swap* genes g and g'. Note that if $g, g' \in M_i$, then M_i will be unchanged.
3. Let $P(\mathbf{M}_N, \mathbf{M}'_N) = \min\{1, \frac{\Phi(\mathbf{M}_N)^{\alpha}}{\Phi(\mathbf{M}'_N)^{\alpha}}\}$.
4. With probability $P(\mathbf{M}_N, \mathbf{M}'_N), \mathbf{M}_{N+1} = \mathbf{M}'_N$, else $\mathbf{M}_{N+1} = \mathbf{M}_N$.

It is easy to see that this chain is ergodic (it is possible to reach any state (collection) from any other state (collection), it is finite, and it is not bipartite) and thus it converges to our desired stationary distribution. We use the parameter α to increase/decrease the difference between $\Phi(\mathbf{M}'_N)$ and $\Phi(\mathbf{M}_N)$ (we used $\alpha = 2$ except where noted). Also, in the second step of the algorithm, we ensure that the number of exclusive alterations is larger than the number of co-occurring by checking that the Dendrix weight $W(M) > 0$. This is to avoid examining sets alterations with high coverage (e.g. altered over 90% of samples) that may have significant exclusivity even though relatively few samples harbor exclusive alterations. We assess convergence of the MCMC algorithm by calculating *total variation distance* of the sampling distributions from multiple chains with different initializations (details below).

Convergence of MCMC from different initial gene sets We assessed the convergence of the MCMC algorithm by comparing the sampling distributions from multiple chains initialized at different starting states. The rationale is that if the multiple chains have converged (i.e. are sampling from the posterior distribution) then the sampling distributions obtained from the different initializations should be very similar. Conversely, if the sampling distributions are different, then one or more of the chains has failed to converge. We computed the distance between the sampling distributions of chains C with different initializations as follows.

First, let $P_c(\mathbf{M})$ be the proportion of iterations in which collection $\mathbf{M} \in \Omega$ was sampled in chain $c \in C$. We compute the total variation distance [72] between the distribution P_c for each chain c to the distribution $P_u(\mathbf{M})$, where u is a chain formed by concatenating the chains in C, defined as

$$||P_c - P_u||_{TV} = \max_{s \in u} \|P_c(s) - P_u(s)\|. \quad (2)$$

A small total variational distance implies that the chain c has converged. We take the mean of the total variation distance across chains.

To assess the convergence of the MCMC algorithm, we ran multiple chains with different initializations. For one of these initializations, we used the collection output by Multi-Dendrix [21] (using the same values of the parameters t and k as in CoMEt). The remaining initializations were random collections. We ran the MCMC algorithm with these initializations, running each chain for a given number of iterations. We consider the chains converged if the mean total variation distance between the chains is smaller than 0.005. Otherwise, we increase the number of iterations by a factor of 1.5. We repeat this process until the chains converge or the total number of iterations per chain reaches a maximum number of iterations, which we set as 1 billion. The output of the MCMC algorithm is the union of the sampling distributions from the different initializations. As an example, Figure S15 shows the results of this procedure on AML mutation data for $t = 3$ and $k = 4$, after 1 million and 10 million iterations. See the main text for details on the convergence parameters we used for simulations and on real data.

Parameter selection

We select δ with the following heuristic procedure. When we run CoMEt with t sets in the collection, ideally we should obtain t cliques in the marginal probability graph. To find the best δ that fulfills the expectation, we search for an "L-corner" in a graph of the number of edges in the marginal probability graph as a function of the edge weight.

More precisely, we first plot a log-log distribution with the number of edges in the marginal probability graph with edge weight $\geq p$ against edge weight p (Figure S16). We choose δ starting from the minimum edge weight p_{min} that contains at least $t \times \binom{k}{2}$ edges in the marginal probability graph. e.g. the yellow horizontal line in Figure S16 shows the number of edges in GBM with $k = 3$ and $t = 3$. We identify a value δ where the number of edges increases dramatically after this value as the probability threshold decreases. To find this value, for each value x we perform a linear regression of two best-fit lines (using root mean squared error) before and after this value. We the first $p > p_{min}$ that forms a "L-corner", i.e. the slope of the two best-fit lines changes from a smaller negative value to a larger negative value as the value x decreases (e.g. moving leftward in Figure S16).

For each TCGA dataset, we ran CoMEt with $\alpha = 2$, $k = 4$ and 100 million iterations using 10 random initializations. We used $t = 3$ for BRCA and $t = 4$ for AML, GBM, and STAD. For BRCA and STAD with subtypes, we ran CoMEt with $k = 4$ and t equal to the number of pre-defined subtypes (4 and 3, respectively), and 100 million iterations using 10 random initializations. See § *Somatic mutation datasets* and Supplementary § *Convergence of MCMC from different initial gene sets* for additional details.

S3 Data

Simulated data

We generated simulated datasets with $t = 1$ implanted pathway using the following approach. Recall $\mathbf{C}$ is a set of highly altered genes whose alterations are not necessarily exclusive.

1. Select k genes to form an "implanted pathway" P.

2. Let γ_P be the fraction of mutated samples in P. Select $\gamma_P \times n$ samples to be exclusively mutated in P, where the proportion of mutations in each gene in P is given by the tuple $\mu_P = (c_1, \ldots, c_k)$.
3. Randomly select samples to be mutated in each gene in $\mathbf{C}$, where the fraction of mutated samples per gene is given by $\gamma_{\mathbf{C}}$.
4. For each of the n samples s in each of the m genes g (including the implanted and cancer genes), mutate g in s with fixed probability q. This step introduces noise into the dataset.

We generated two different versions of the simulated datasets, depending on whether we implanted a single or multiple gene sets. We describe the method for generating datasets with multiple implanted gene sets in the main text. For all simulated datasets, we used $n = 500$, $|\mathbf{C}| = 5$, $\gamma_{\mathbf{C}} = (0.67, 0.49, 0.29, 0.29, 0.2)$, and $q = 0.0027538462$.[1] We used $\mu_P = (0.5, 0.35, 0.15)$ for the single pathway simulations.

S4 Supplementary Figures

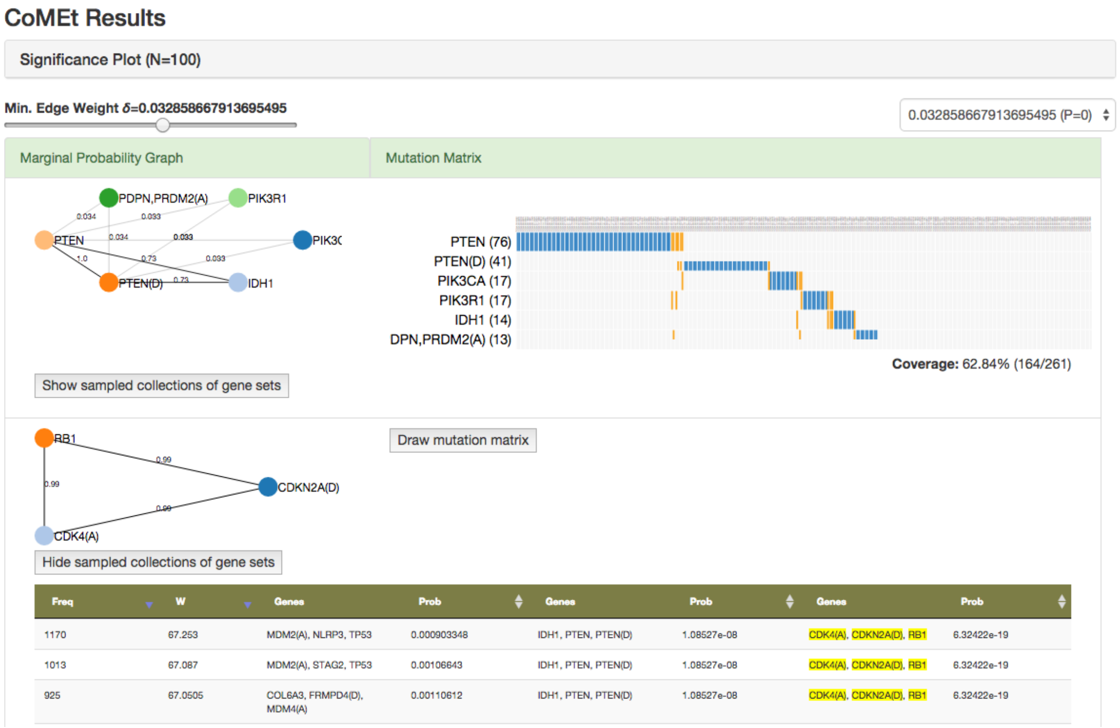

Figure S1: **Screenshot of the web application for interactive visualization of CoMEt results.**

[1]We chose values for $\mathbf{C}$ and q using values calculated from real data. We choose C to match the mutation frequencies of the five most mutated genes in the TCGA glioblastoma dataset. We calculated q empirically from the TCGA breast cancer mutation matrix.

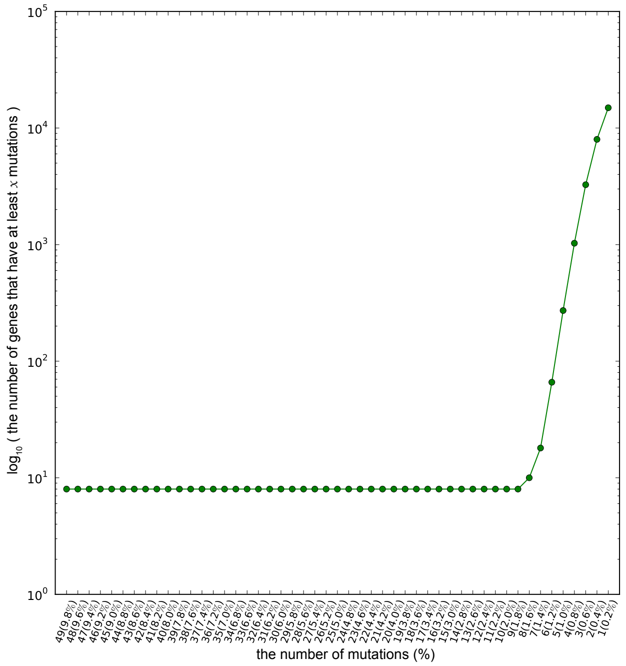

Figure S2: **The distribution of the number of genes with $\geq x$ mutations in simulated data.** We removed those genes mutated in fewer than 1% of mutations, i.e. genes mutated in fewer than 5 samples.

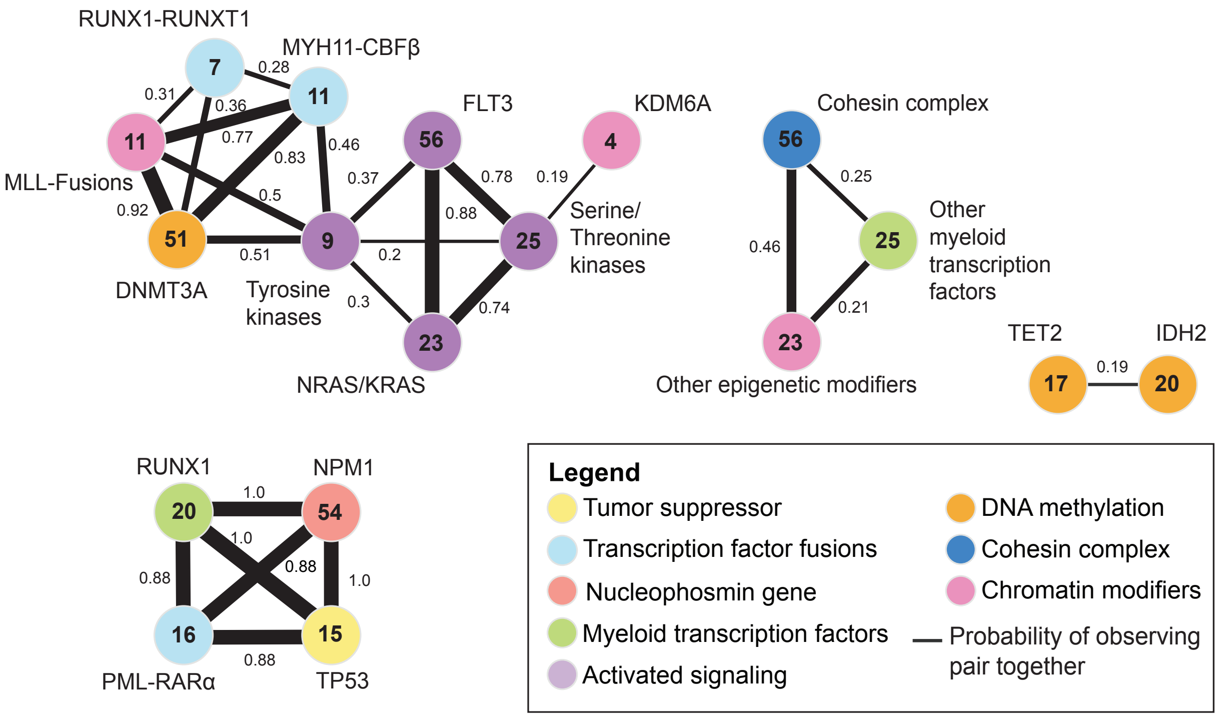

Figure S3: **CoMEt results on AML dataset with $t = 4$ and $k = 4$.**

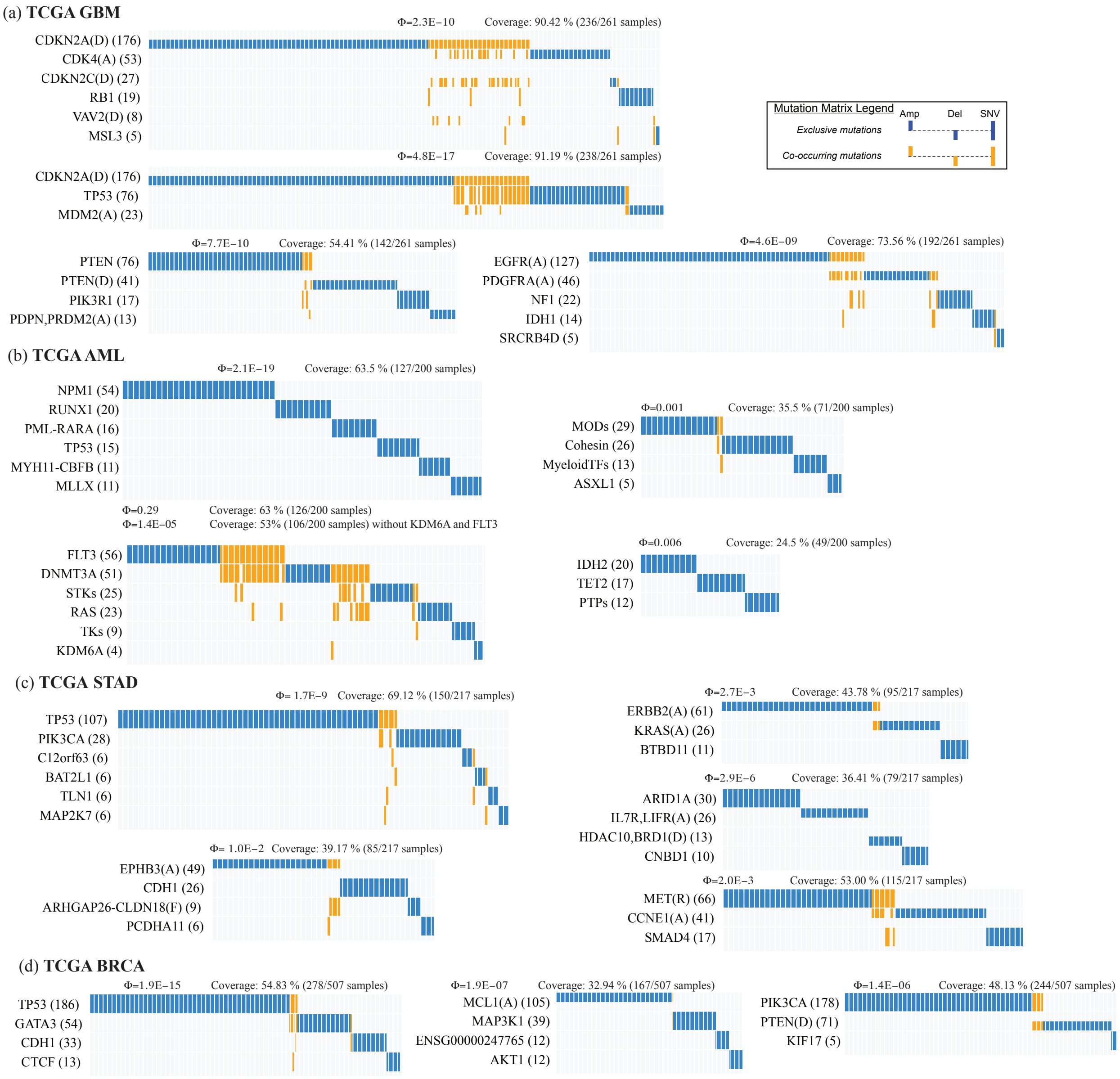

Figure S4: **Mutation matrices for the CoMEt results on (a) TCGA GBM, (b) TCGA AML, (c) TCGA STAD, and (d) TCGA BRCA datasets.** The matrices have alterations as rows, and samples as columns. Each cell indicates whether or not an alteration occurred in a particular sample, where grey indicates the sample was not altered. Samples with co-occurring alterations in the same set are colored orange, while exclusive alterations are colored blue.

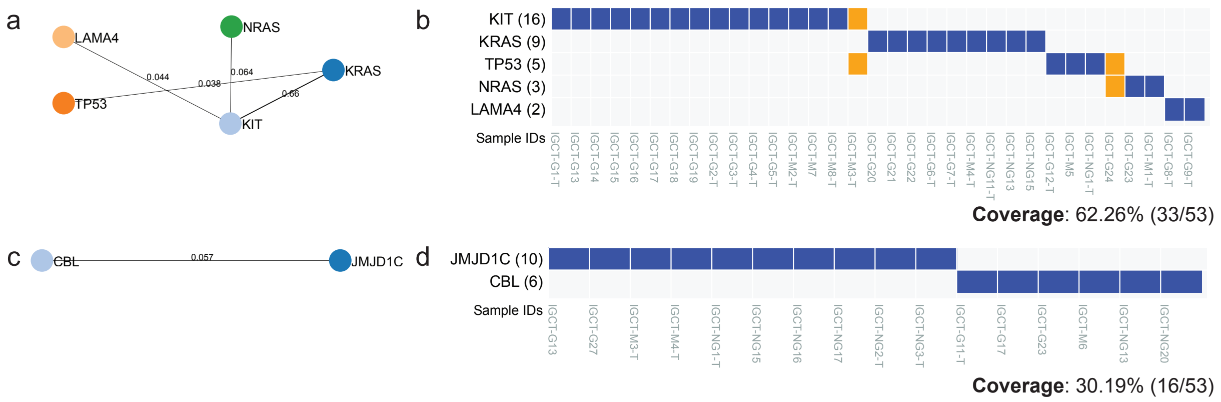

Figure S5: **Statistically significant modules identified by CoMEt (with $k = 3$, $t = 1$) on ICGTs from Wang *et al.* [30].** (a,c) Marginal probability graphs of the two modules. (b,d) Mutation matrices of the two modules. Representation as in Figure 4.

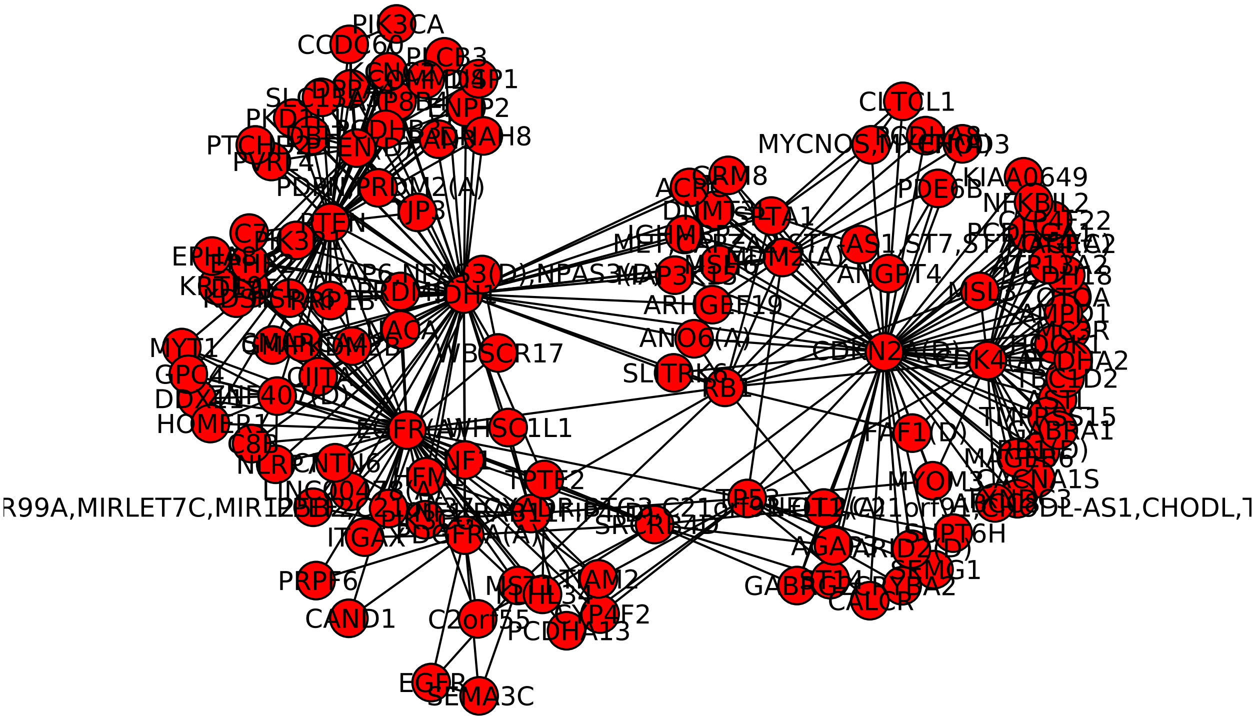

Figure S6: mutex results on the TCGA GBM dataset from Leiserson *et al.* [21].

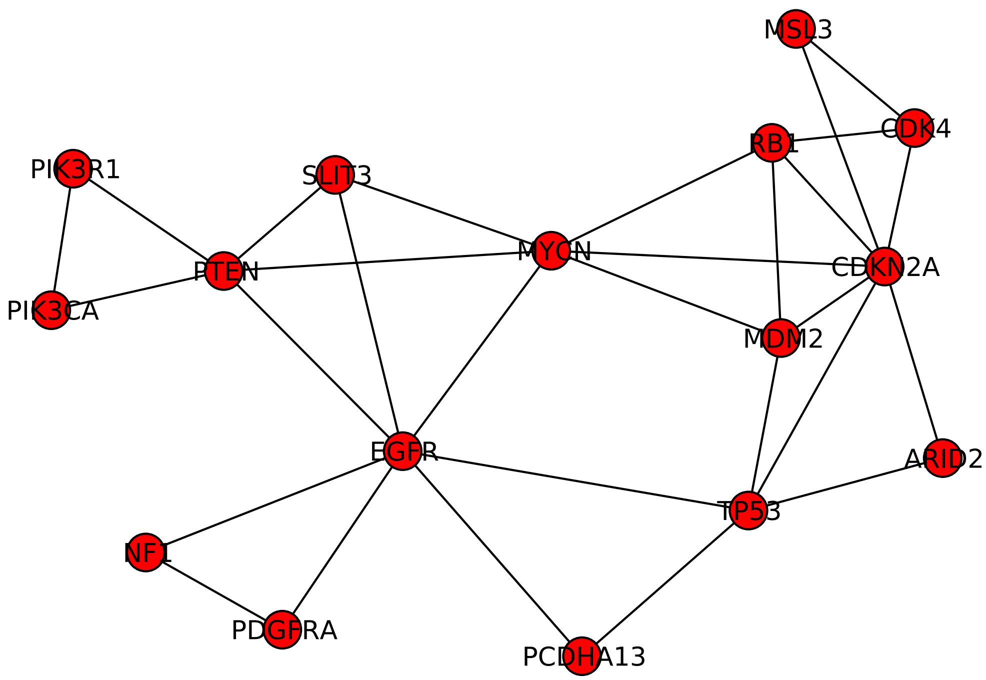

Figure S7: mutex results on the TCGA GBM dataset from Leiserson *et al*. [21] with mutex's default signaling network.

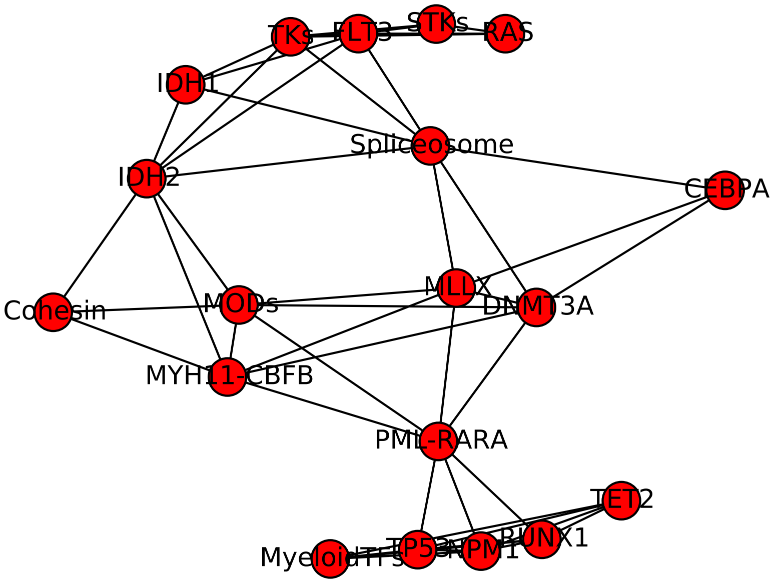

Figure S8: Multi-Dendrix results on the TCGA AML dataset [3].

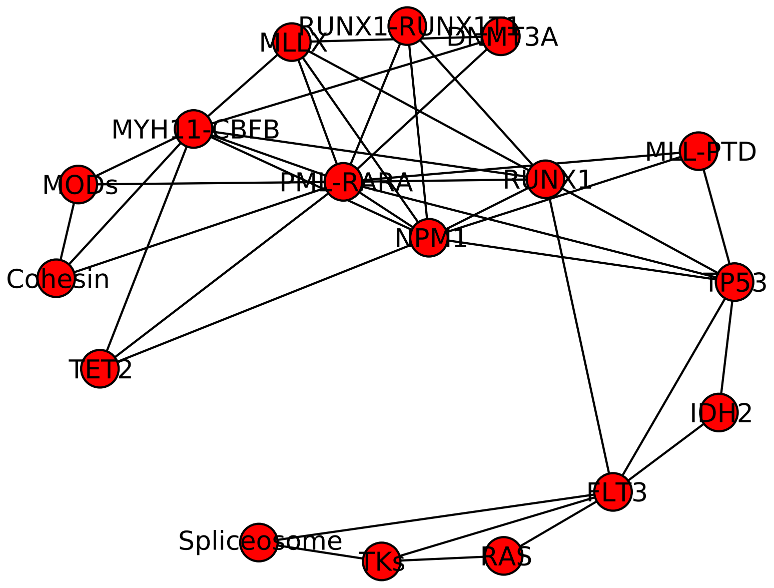

Figure S9: mutex results on the TCGA AML dataset [3].

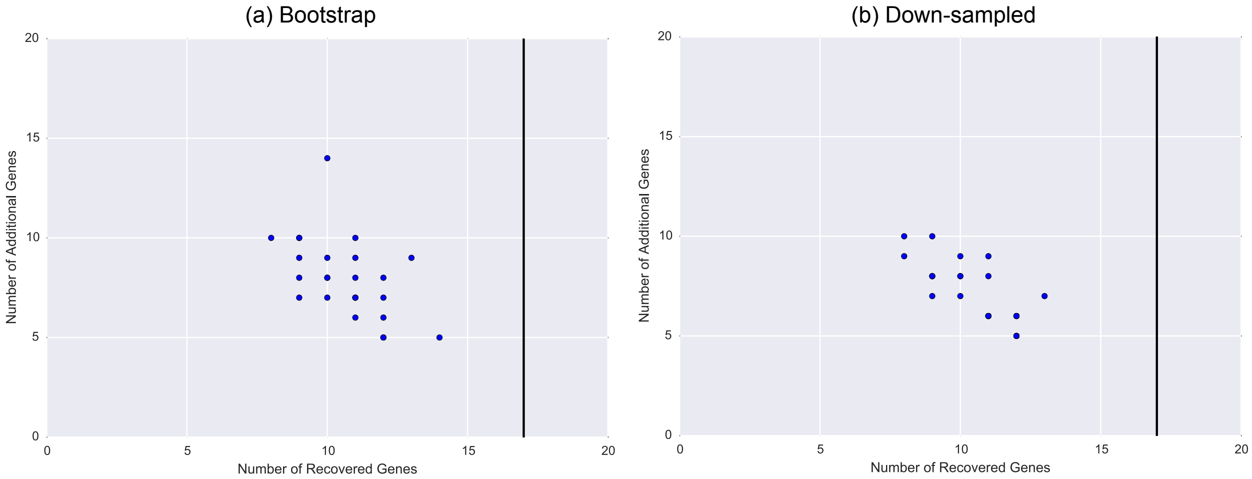

Figure S10: Robustness of CoMEt on TCGA GBM [1] datasets from Leiserson *et al.* [21]. Datasets were (a) bootstrapped and (b) down-sampled to include only 50% of the samples. We ran CoMEt on 25 such datasets, and computed the number of recovered genes (x-axis) and number of additional genes (y-axis) compared to the CoMEt results on the full dataset. For comparison, the CoMEt results on the full dataset included 17 genes (black line).

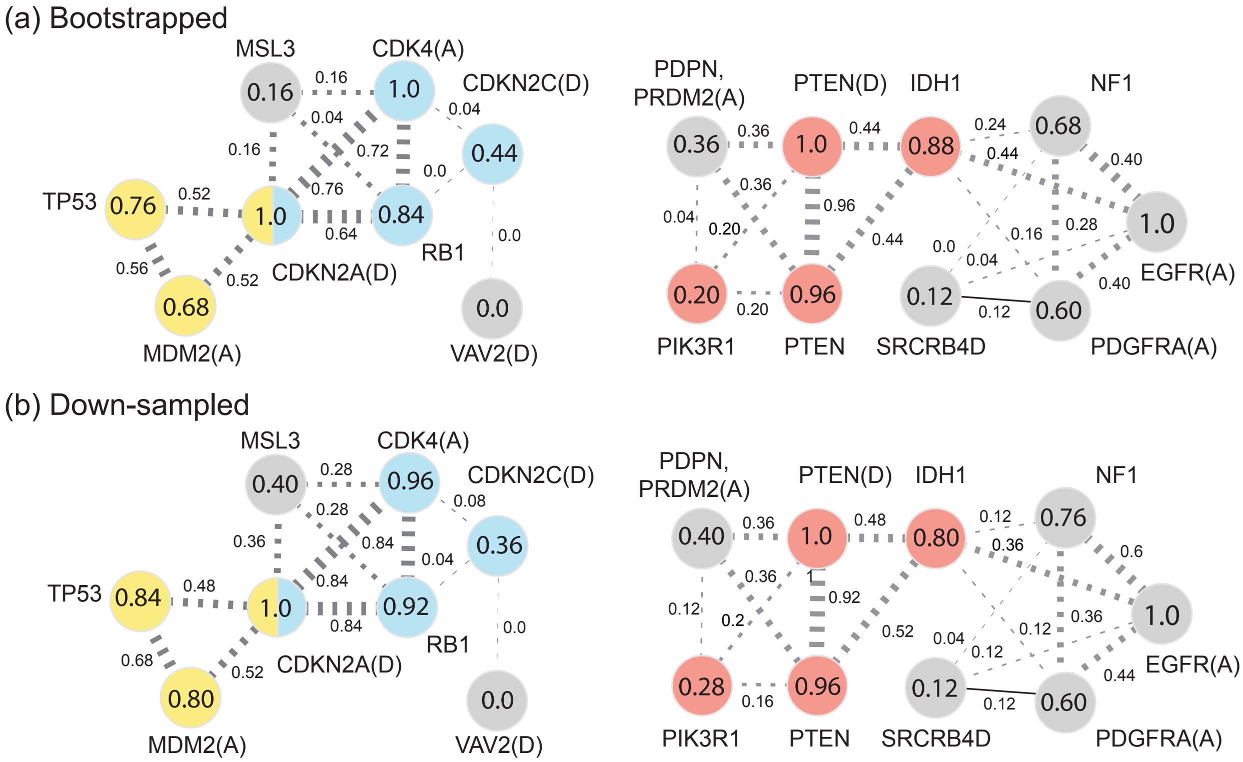

Figure S11: **Robustness of the modules identified by CoMEt on the TCGA GBM [1] dataset from Leiserson *et al.* [21]**. We ran CoMEt on TCGA GBM datasets (a) bootstrapped (sampled with replacement) and (b) down-sampled to include only 50% of the samples. Shown is the marginal probability graph output by CoMEt on the TCGA GBM dataset. Nodes and edges are labeled with the proportion of down-sampled datasets in which they were identified by CoMEt. The most mutated genes in each of the Rb, p53, and PI(3)K signaling pathways were identified on at least 68% (17/25) and 80% (20/25) of the bootstrapped and down-sampled datasets, respectively.

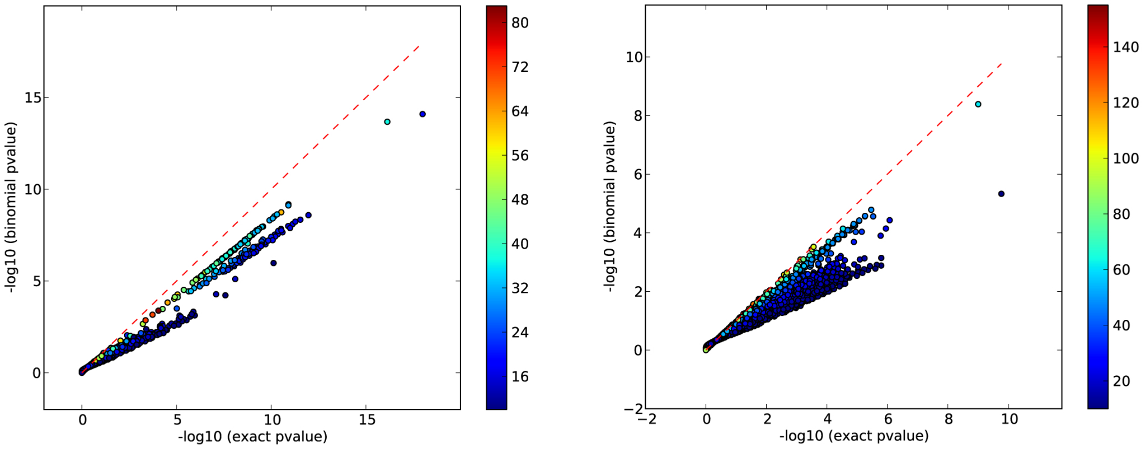

Figure S12: **Scatter plot between negative log of exact and binomial P-values for all sets of $k = 3$ alterations on the GBM dataset (left) and BRCA dataset (right).** The color of each dot represents the number of co-occurring alterations according to the scale at the right. Note that the P-values for the exact test much smaller than the binomial only in cases with relatively low number of co-occurrences. These cases are the fastest to compute with the tail enumeration algorithm.

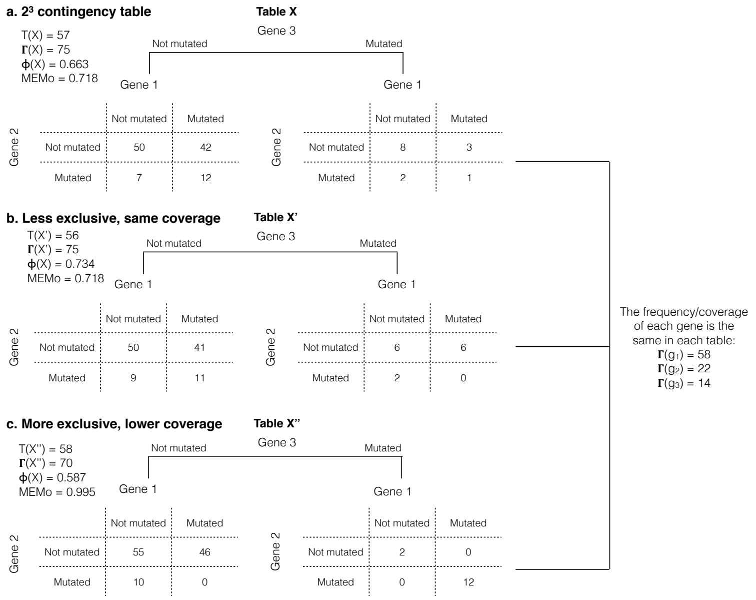

Figure S13: **Two cases where the MEMo permutation test statistic Γ (the coverage, or number of altered samples) deflates or inflates the P-value compared to the CoMEt test statistic T (the number of samples with exclusive mutations).**

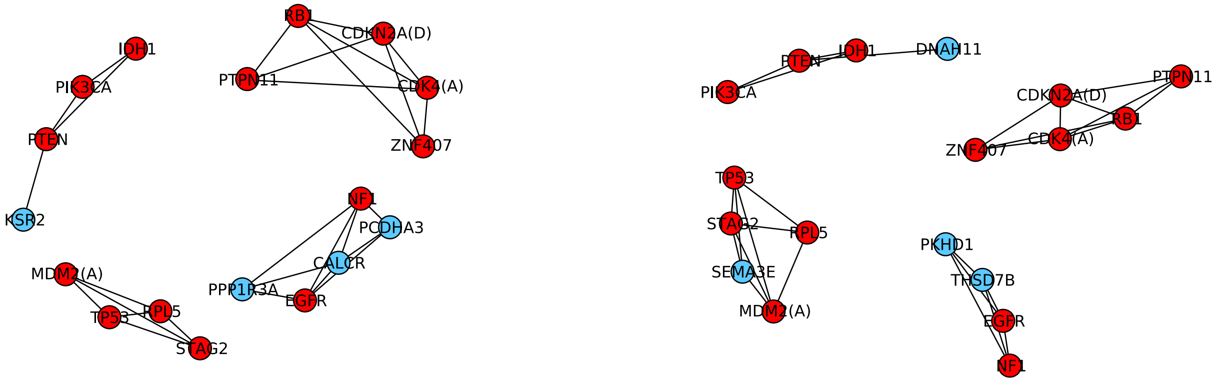

(a) CoMEt

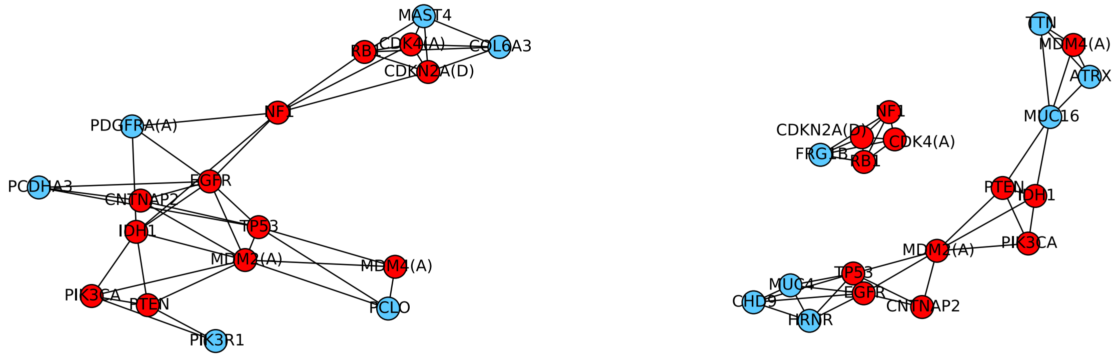

(b) Multi-Dendrix

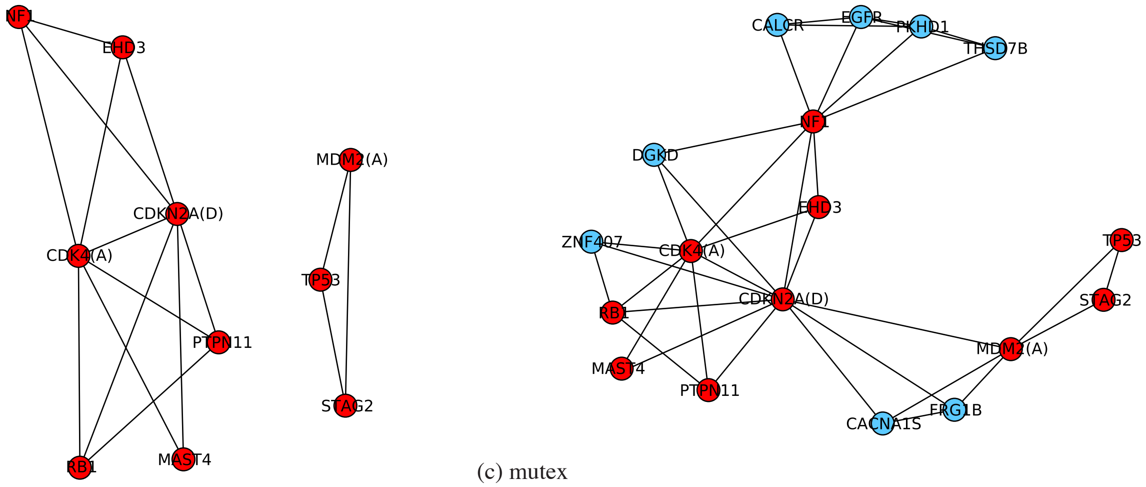

(c) mutex

Figure S14: (a) CoMEt modules, (b) Multi-Dendrix consensus, and (c) Mutex groups from the TCGA Pan-cancer GBM datasets [5] with (left) and without (right) mutation filtering with the MutSigCV algorithm. Red and blue circles represent genes that are common and different between the two results, respectively.

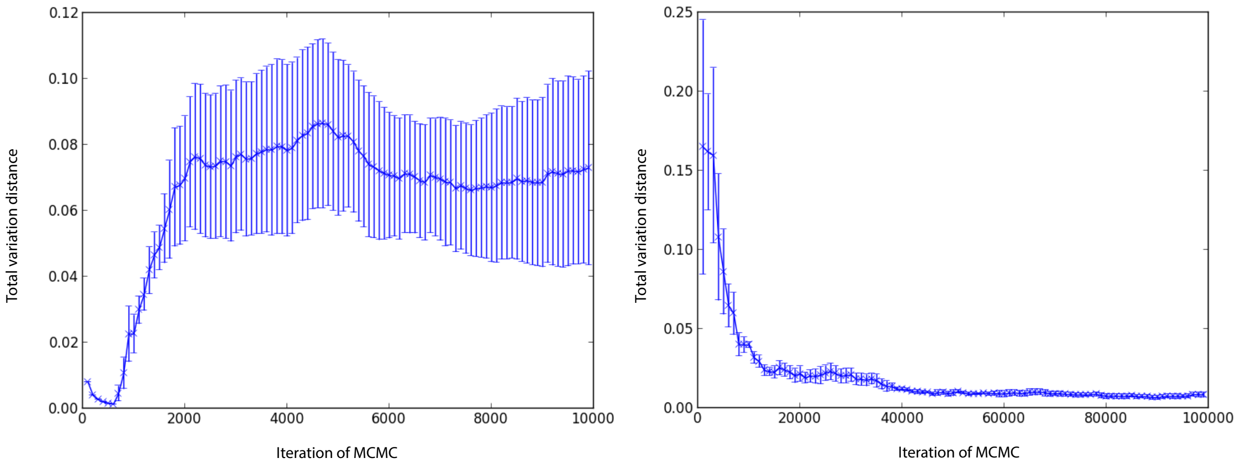

Figure S15: **Plots of the total variation distance distribution in each iteration for an MCMC run with $1M$ iterations (left) and an MCMC run with $10M$ iterations (right) on AML mutation data for $t = 3$ and $k = 4$.**

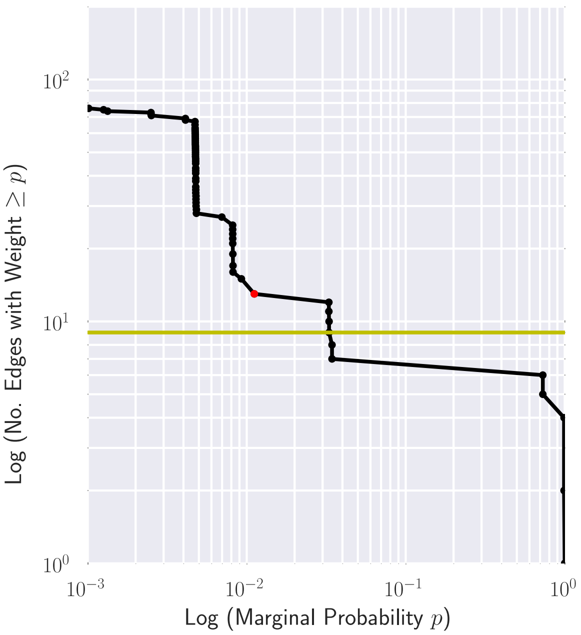

Figure S16: **The distribution of the number of edges with weight $\geq p$ in GBM with $k = 3$ and $t = 3$ in log-log scale.** The red dot indicates the first hitting edge weight where the change in slope is negative (when moving leftward) such that the number of edges in the subgraph is at least $t \times \binom{k}{2} = 9$ (as the horizontal yellow line).

Supplementary References

71. Forbes, S.A., Bindal, N., Bamford, S., Cole, C., Kok, C.Y., *et al.*: COSMIC: mining complete cancer genomes in the Catalogue of Somatic Mutations in Cancer. Nucleic acids research **39**(Database issue), 945-50 (2011). doi:10.1093/nar/gkq929

72. Tierney, L.: Markov chains for exploring posterior distributions. The annals of statistics **22**(4), 1701-1728 (1994).
